# Synthesis of high-titer alka(e)nes in *Yarrowia lipolytica* is enabled by a discovered mechanism

**DOI:** 10.1038/s41467-020-19995-0

**Published:** 2020-12-03

**Authors:** Jingbo Li, Yongshuo Ma, Nian Liu, Bekir E. Eser, Zheng Guo, Peter Ruhdal Jensen, Gregory Stephanopoulos

**Affiliations:** 1 Department of Chemical Engineering, Massachusetts Institute of Technology, Cambridge, MA 02142 USA; 2 Department of Engineering, Aarhus University, Gustav Wieds Vej 10, Aarhus, 8000 Denmark; 3 National Food Institute, Technical University of Denmark, Kongens Lyngby, 2800 Denmark

**Keywords:** Metabolic engineering, Metabolic engineering, Applied microbiology

## Abstract

Alka(e)nes are ideal fuel components for aviation, long-distance transport, and shipping. They are typically derived from fossil fuels and accounting for 24% of difficult-to-eliminate greenhouse gas emissions. The synthesis of alka(e)nes in *Yarrowia lipolytica* from CO_2_-neutral feedstocks represents an attractive alternative. Here we report that the high-titer synthesis of alka(e)nes in *Yarrowia lipolytica* harboring a fatty acid photodecarboxylase (*Cv*FAP) is enabled by a discovered pathway. We find that acyl-CoAs, rather than free fatty acids (FFAs), are the preferred substrate for *Cv*FAP. This finding allows us to debottleneck the pathway and optimize fermentation conditions so that we are able to redirect 89% of acyl-CoAs from the synthesis of neutral lipids to alka(e)nes and reach titers of 1.47 g/L from glucose. Two other CO_2_-derived substrates, wheat straw and acetate, are also demonstrated to be effective in producing alka(e)nes. Overall, our technology could advance net-zero emissions by providing CO_2_-neutral and energy-dense liquid biofuels.

## Abstract (web summary)

Alka(e)nes with chain lengths in C5-C23 range are ideal fuel components. Here, the authors report that high-titer production of alak(e)nes in pathway engineered *Yarrowia lipolytica*, which is enabled by the finding that acyl-CoA is another substrate of fatty acid photodecarboxylase (FAP).

## Introduction

Increasing greenhouse gas (GHG) emissions is the leading cause of climate change^1^ and efforts to minimize the use of fossil fuels have mainly focused on deploying alternative energy sources such as bioethanol and electrical power. However, due to their low energy densities, both of these approaches struggle to transform the aviation, trucking, and shipping industries, making long-distance transport particularly difficult to decarbonize^2^. It has been reported that air transportation and other modes of heavy-duty transport emitted 830 and 1060 Mt of CO_2_ in 2014, respectively, accounting for 24% of difficult-to-eliminate GHG emissions, and these numbers are projected to increase in the foreseeable future^2^. Although up to 0.8% reduction of overall emissions in the aviation industry could be achieved by blending conventional fuels with 1% of bio-jet fuels^3^, bio-jet fuels such as bio-kerosene are currently derived from vegetable oils, intensifying concerns over competition with food supply^4^. Therefore, the production of alka(e)nes with chain lengths in the C5-C23 range from sugar, biomass, or CO_2_-derived feedstocks could have better potential in disrupting the current state of transportation, leading to superior mitigation of CO_2_ emissions. Generally, renewable alka(e)nes can be synthesized either chemically or biologically. The former typically involves sequential hydrolysis and dehydration of biomass followed by C–C bond formation and hydrodeoxygenation, which require elevated temperatures and pressures, as well as different types of catalysts, complicating the overall process^5^. By contrast, bioconversion approaches offer high carbon- and energy-conversion efficiencies in single-unit operations, yielding an overall simplified process^6^.

Three classes of hydrocarbons can be biologically synthesized: those derived from fatty acids, isoprenoids, and polyketides, respectively^4^. For fatty acid-derived hydrocarbons, two major metabolic pathways have been identified, the two-step cyanobacterial pathway involving acyl-ACP reduction and aldehyde decarbonylation^7^, and the one-step decarboxylation reaction from free fatty acids (FFAs)^8–11^. In prior studies, both pathways were harnessed in *E. coli* to synthesize alka(e)nes with varying chain lengths, with reported titers ranging from 1.3 to 1310 mg/L and corresponding yields ranging from 0.43 to 11 mg/g glucose (Supplementary Table 1). Another model microorganism, *Saccharomyces cerevisiae*, was engineered to produce alka(e)nes as well, albeit achieving relatively lower titers and yields compared to those obtained with *E. coli* (Supplementary Table 1). *Yarrowia lipolytica*, a widely recognized model oleaginous organism^12^ due to its high lipid synthesis capacity, was also used and shown to be able to produce 23 mg/L alka(e)nes through the cyanobacterial pathway^13^. Very recently, a fatty acid photodecarboxylase from *Chlorella variabilis* NC64A (*Cv*FAP) was discovered to decarboxylate FFAs with preference to palmitic and heptadecanoic acids over lauric, myristic, stearic, oleic, and linoleic acids^11^. It was then expressed in an FFA-overproducing *Y. lipolytica* strain^14^. Although the production of 10.87 mg/L and 58.7 mg/L alka(e)nes were achieved in the presence of light with batch and fed-batch fermentation modes, respectively, no information regarding the responsible metabolic pathways was detailed^14^. Furthermore, it is unknown as to why adding crude cell lysate to the purified enzyme accelerates palmitic acid to pentadecane conversion in vitro^15^. In order to study how *Cv*FAP functions and uncover its relations to alka(e)ne synthesis in *Y. lipolytica*, an oleaginous yeast that produces approximately 30 mg/L FFAs before metabolic rewiring^16^, in this work, we set out to harness the photodecarboxylation pathway as well as its supporting pathways in *Y. lipolytica*. By identifying acyl-CoAs as one of the substrates for *Cv*FAP, we are able to significantly improve the decarboxylation flux such that 89% of acyl-CoAs are directed away from the synthesis of neutral lipids and to alka(e)nes under optimal conditions. Additionally, our strain is compatible with two CO_2_ fixation schemes, one based on biomass hydrolysates and the other on CO_2_-derived acetic acid, both of which can be fed to *Y. lipolytica* directly for alka(e)ne production.

## Results

### Engineering and rewiring pathways for alka(e)ne synthesis in *Y. lipolytica*

Expression of the codon-optimized Cv*FAP* in *Y. lipolytica* po1f resulted in YLjbl-2 which produced 15.3 mg/L alka(e)nes. The constituents were verified to be heptadecane (C17:0), 8-heptadecene (C17:1), 6,9-heptadecadiene (C17:2), and pentadecane (C15:0) by comparing their mass spectra (Supplementary Fig. 1) to a NIST library as well as a previous report^17^.

**Fig. 1:**
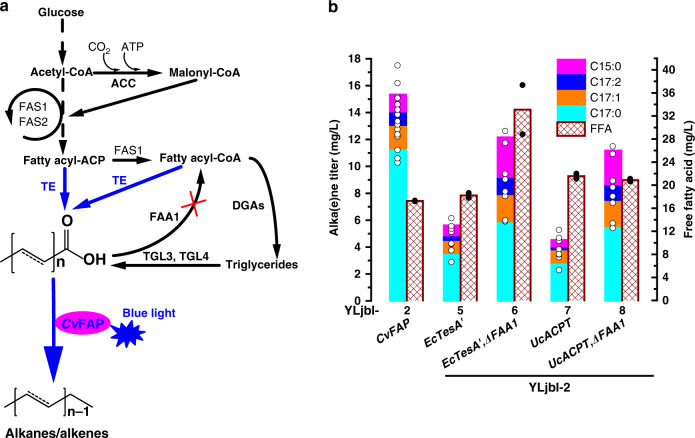
The effect of expression of thioesterases on alka(e)ne production. **a** Metabolic pathways for alka(e)ne production employing the fatty acid photodecarboxylase according to previous literature that free fatty acids are the precursors of alka(e)nes (the revised pathway is shown in Fig. 2). Black arrows represent native pathways while blue arrows indicate heterologous pathways. TE, acyl-CoA/acyl-ACP thioesterases; TGL3 and TGL4, intracellular lipases from *Y. lipolytica*; FAA1, acyl-CoA synthase, ACC, acetyl-CoA carboxylase, FAS1 and FAS2, fatty acid synthase 1 and 2, DGAs, diacylglycerol transferases. **b** Alka(e)ne production is observed by expressing photodecarboxylase in a wild-type strain. Expression of thioesterases (*Ec*TesA′ and *Uc*ACPT) and knockout of *FAA1* did not enhance alka(e)ne production but decreased, although free fatty acid titers were increased. Fermentations were performed in 50 mL conical shake flasks with a working volume of 13 mL and an initial OD_600_ of 0.1 for 3 days in the dark followed by 3 days in blue light generated by light source 1 (Supplementary Fig. 16). Fermentation medium was composed of 20 g/L glucose, 6.9 g/L yeast nitrogen base (without amino acids), and 1 g/L yeast extract. Data represent mean value, *n* = 2 biologically independent samples. Source data underlying Fig. 1b are provided as a Source Data file.

Since FFAs were believed to be the substrate of *Cv*FAP in in vitro studies^11,15^, harnessing FFA-producing pathways and blocking FFA-consuming pathways in YLjbl-2 were expected to improve alka(e)ne synthesis. To investigate this hypothesis, we expressed enzymes for FFAs synthesis including the bifunctional acyl-ACP/acyl-CoA thioesterase (*Ec*TesA′) from *E. coli* and the acyl-ACP thioesterase from *Umbellularia californica* (*Uc*ACPT)^13,18^ in strain YLjbl-2. However, the thioesterase expression depressed alka(e)ne synthesis, though FFA titers were enhanced (Fig. 1b). We then disrupted FAA1, the cytosolic enzyme responsible for converting FFAs to acyl-CoAs^19^, in strains overexpressed thioesterases. Compared with strains overexpressed thioesterase, *FAA1* knockout improved the alka(e)ne titer and FFA titer in both cases. However, compared with YLjbl-2, neither the thioesterase expression nor the *FAA1* knockout improved alka(e)ne synthesis, though FFA titers were enhanced (Fig. 1b). Together with *FAA1* knockout, we also expressed the FAS-*Ec*TesA′ and FAS-*Uc*ACPT fusion proteins, which were found to be effective for FFA production in *Y. lipolytica*^13^, but the resulting strains produced less alka(e)nes as well (Supplementary Fig. 2). Targeting both thioesterases to the endoplasmic reticulum where triglycerides are synthesized increased FFA formation but did not enhance alka(e)ne production either (Supplementary Fig. 2). Expression of lipases^20^ in YLjbl-2 was also carried out, but no increases in alka(e)ne titers were observed despite the improved FFA production (Supplementary Fig. 2). We also noticed that the expression of the *Cv*FAP in an FFA-overproducing strain that was able to produce around 2 g/L intracellular and 2.7 g/L extracellular FFAs^20^ gave rise to 10.87 mg/L alka(e)nes from 50 g/L glucose^14^, which is lower than the titer produced by YLjbl-2 from 20 g/L glucose (Fig. 1b). These results suggest that the overproduction of FFAs is not a prerequisite for obtaining more alka(e)nes. Together with the observation that the purified *Cv*FAP enzyme showed 6–12 times lower activity than the crude cell lysate in an earlier report^15^, we speculate that there may be an unknown mechanism regarding the *Cv*FAP enzyme when converting FFAs to alka(e)nes.

### A proposed metabolic pathway for alka(e)ne synthesis in *Y. lipolytica*

**Fig. 2:**
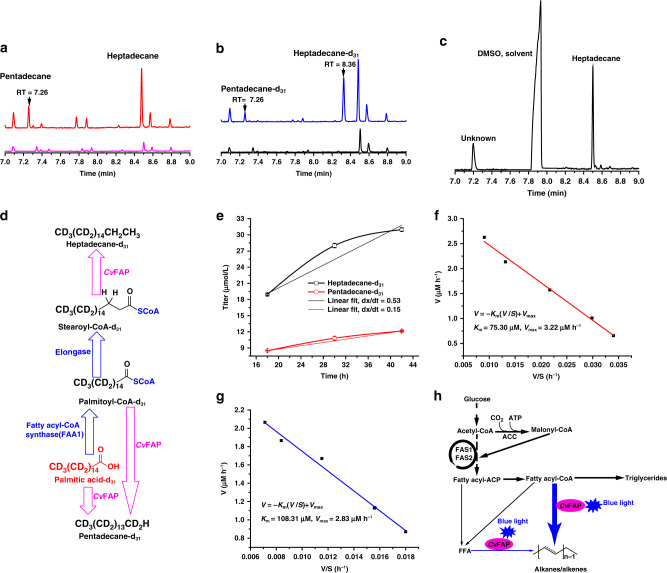
A proposed metabolic pathway for alka(e)ne synthesis in *Y. lipolytica*. **a**, **b** GC chromatograms from strain YLjbl-2-Δ*FAA1* (red) and strain YLjbl-2 (blue) cultivated with exogenous palmitic acid-d_31_ supplementation, respectively. Magenta and black lines represent the same cultivation without palmitic acid-d_31_ addition. Two compounds with retention time of 7.26 and 8.36 min were found and identified to be pentadecane-d_31_ and heptadecane-d_31_, respectively. **c** GC chromatogram of products from the in vitro photocatalytic reaction using stearoyl-CoA lithium salt (≥90%, Sigma-Aldrich) as the substrate and purified *Cv*FAP as the catalyst, showing that heptadecane could be synthesized from stearoyl-CoA. **d** Proposed mechanism for pentadecane-d_31_ and heptadecane-d_31_ formation from exogenous palmitic acid-d_31_. Blue arrows indicate endogenous pathways while magenta arrows represent photodecarboxylation. **e** In vivo pentadecane-d_31_ and heptadecane-d_31_ formation rates resulting from the strain YLjbl-2 indicated that fatty acyl-CoA might be the preferred substrate for the photodecarboxylase. **f**, **g** Kinetic analyses of purified *Cv*FAP at fixed concentrations for the determination of *K*_m_ and *V*_max_ when stearoyl-CoA lithium salt and lithium stearate (≥98.0%, TCI America) were used as substrates, respectively. Values of *K*_m_ and *V*_max_ were determined through Eadie-Hofstee plots. The results suggested that fatty acyl-CoAs were the preferred substrate for *Cv*FAP over free fatty acids. **h**: A proposed pathway for alka(e)ne production catalyzed by *Cv*FAP. Blue arrow represents photodecarboxylation and bold blue arrow indicates higher metabolic flux. ACC, acetyl-CoA carboxylase, FAS1 and FAS2, fatty acid synthase 1 and 2. Data represent mean value, *n* = 2 biologically independent samples. Source data underlying Fig. 2e–g are provided as a Source Data file.

In order to explore the mechanism, we designed experiments where labeled palmitic acid-d_31_ was metabolized by YLjbl-2 and YLjbl-2-Δ*FAA1*. Both strains produced pentadecane-d_31_ (C15:0) directly from palmitic acid-d_31_ through decarboxylation (Fig. 2a, b, Supplementary Fig. 3a). We also detected stearic acid methyl ester-d_31_ in methylated samples from YLjbl-2 cells but not from YLjbl-2-Δ*FAA1* cells (Supplementary Fig. 3b). Since the CoA form is required for acyl chain elongation from C16 to C18, this confirms the existence of stearoyl-CoA-d_31_ in YLjbl-2 cells.

Interestingly, a formed compound with the retention time of 8.36 min was observed by supplementing YLjbl-2 with palmitic acid-d_31_ (Fig. 2b). We further identified the peak through GC-MS and confirmed it to be heptadecane-d_31_ (C17:0) according to its molecular weight and MS fragments (Supplementary Fig. 3c). Since this product comes from the decarboxylation of a C18 acyl chain and no stearic acid-d_31_ was detected (Supplementary Fig. 3d), we hypothesized that stearoyl-CoA-d_31_ could be the precursor for synthesizing heptadecane-d_31_ by *Cv*FAP. In order to provide evidence for this hypothesis, we purified the enzyme and subjected it to stearoyl-CoA lithium salt as the substrate and verified the formation of heptadecane-d_31_ (C17:0) (Fig. 2c, Supplementary Fig. 3e). Based on these results, we proposed the full pathway where pentadecane-d_31_ (C15:0) can be synthesized from both palmitic acid-d_31_ and palmityl-CoA-d_31_, while heptadecane-d_31_ (C17:0) can only be derived from stearoyl-CoA-d_31_ in *Y. lipolytica* as shown in Fig. 2d.

To determine whether FFA or acyl-CoA is the preferred substrate, we examined the titers of pentadecane-d_31_ and heptadecane-d_31_ over time in vivo and calculated their respective cumulative formation rates (Fig. 2e). Our measurements showed that the titer and the cumulative formation rate of heptadecane-d_31_ (C17:0) were 2-fold and 3.5-fold higher than those of pentadecane-d_31_ (C15:0), respectively. Together with our proposed pathway in Fig. 2d, it seems that *Cv*FAP prefers acyl-CoA over FFA. In order to confirm this, we studied the enzyme kinetics of *Cv*FAP using stearoyl-CoA lithium salt and lithium stearate as substrates. The enzyme displayed Michaelis–Menten kinetics with a *K*_m_ constant of 75.30 or 108.31 μM, a *V*_max_ of 3.22 or 2.83 μM/h, and a *k*_cat_ of 1114.62 and 979.62 s^−1^ when the substrate was stearoyl-CoA or stearic acid, respectively (Fig. 2f, g). Thus, specificity constant (*k*_cat_/*K*_m_) for stearoyl-CoA is about 1.6-fold higher compared to specificity constant for stearic acid (14.8 vs 9.0), providing further evidence that acyl-CoA is the preferred substrate for *Cv*FAP.

We further proposed a catalytic mechanism as illustrated in Supplementary Note 1. In the first step, acyl-CoA binds to the active site and gets hydrolyzed with the help of the polar residues and water/hydroxide around thioester bond (Supplementary Fig. 4). In the next step, CoA dissociates from the active site, whereas FFA remains bound inside the hydrophobic substrate tunnel. This is followed by binding of FAD into the region of the active site that has been abandoned by CoA. Thereafter the light-dependent mechanism takes place through FAD-dependent radical chemistry leading to decarboxylation of FFA into alkane, as described earlier^11^. Docking of palmitoyl-CoA into the *Cv*FAP structure indicates the presence of multiple polar side chains around thioester bond, which can possibly support a hydrolysis mechanism. Given the diversity of active site motifs present in a wide variety of thioesterases^21^, further mechanistic and structural studies will be required to reveal the exact nature of the hydrolysis step.

Our proposed mechanism, where a larger substrate is hydrolyzed before it goes through complex chemistry in a single enzyme active site, is not uncommon in literature^22^. CalE7, a bacterial thioesterase that plays a role in enediyne biosynthesis, first hydrolyzes the thioester bond of its ACP-tethered acyl substrate before catalyzing decarboxylation of the resulting β-ketocarboxylic acid intermediate^22^. An active site arginine acting as an oxyanion hole and water or a hydroxide acting as nucleophile have been proposed to catalyze the thioester hydrolysis step. Another literature example is thiazole biosynthetic enzyme, Thi4, present in yeast and archaea. This enzyme firstly hydrolyzes its substrate NAD into nicotinamide and ADP-ribose. Afterwards, ADP-ribose goes through a complex transformation that involves binding of iron and other substrates into the active site to carry out multiple steps including iron-dependent sulfur transfer^23^. Analogous to *Cv*FAP, Thi4 can also take ADP-ribose as a substrate instead of NAD (acyl-CoA vs. FFA).

These findings support the proposed metabolic pathway for alka(e)ne synthesis when the photodecarboxylase is used (Fig. 2h). It indicates that acyl-CoA is the primary precursor for producing alk(e)nes in *Y. lipolytica* due to its abundant availability and and its higher specificity constant towards *Cv*FAP. Thus, improvements to the acyl-CoA pool or *Cv*FAP efficiency may further increase alka(e)ne titers.

### Debottlenecking alka(e)ne synthesis by increasing the copy number of Cv*FAP*

Having established that acyl-CoA is the preferred substrate of *Cv*FAP, we sought to increase its availability by knocking out *DGA1* and *DGA2*, which encode enzymes that are responsible for sequestering acyl-CoAs in triglycerides. However, both single and double deletions resulted in lower alka(e)ne and lipid titers relative to the parental strains (Supplementary Fig. 5a). It was shown in a previous study^24^ that acyl-CoA intermediates function as feedback inhibitors to fatty acid synthesis, and suppressed fatty acid synthesis further resulted in growth defects^25^. Deletion of *DGA1*, *DGA2*, or both decreased the overall pool of acyl-CoA, which led to lower availability of precursors for alka(e)ne synthesis. Additionally, we combined Δ*DGA1* and/or Δ*DGA2* with Cv*FAP* overexpression via introducing the linearized Cv*FAP* plasmid once more. This combination of gene modulations produced 1.5 to 3-fold more alka(e)nes than the corresponding parental strains (Supplementary Fig. 5b). Lipid titer, on the contrary, decreased slightly, possibly due to the diversion of acyl-CoA away from triglycerides to alka(e)nes. Therefore, under the same acyl-CoA flux, higher *Cv*FAP activity drove more acyl-CoA to alka(e)nes, which also proved our finding that acyl-CoA was one of the precursors for *Cv*FAP. Overall, these results support the effectiveness of increasing decarboxylation rate by increasing the Cv*FAP* copy number.

**Fig. 3:**
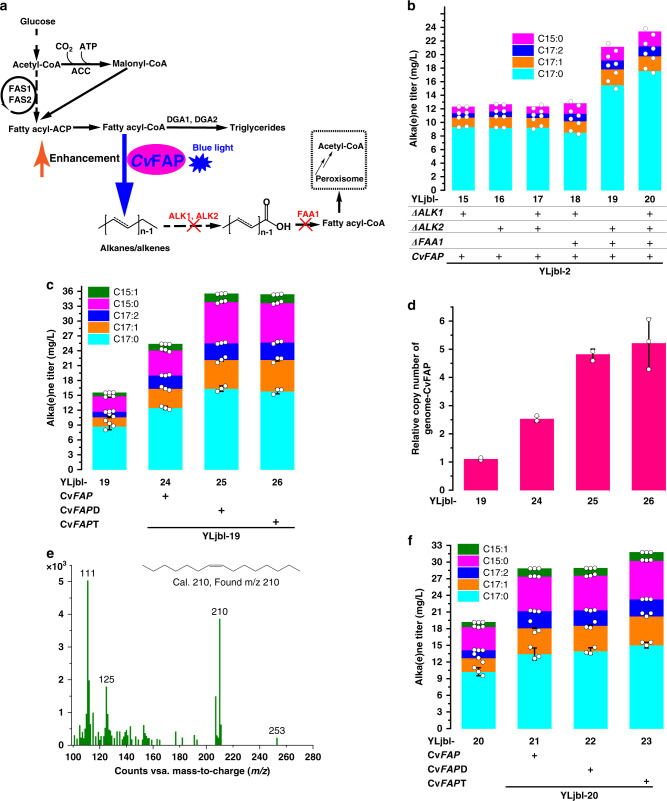
Increasing CvFAP copy number debottlenecked the pathway for alka(e)ne production. **a** Metabolic pathways for improving alka(e)ne production by increasing Cv*FAP* copy number. Abbreviations: ACC, acetyl-CoA carboxylase; FAS1 and FAS2, fatty acid synthase 1 and 2; DGAs, diacylglycerol transferases; ALK1 and ALK2, cytochrome P450ALKs belonging to the CYP52 family with major functions in n-alkane assimilation;^46^ FAA1 was found to be related to n-alkane assimilation^19^. **b** Overexpression of Cv*FAP* in strains with double deletions of *FAA1* and *ALK2* and triple deletions of *FAA1*, *ALK1*, and *ALK2* enhanced alka(e)ne synthesis more profoundly (*n* = 2 biologically independent samples). **c** Compared with the parental strain YLjbl-19, Cv*FAP*, Cv*FAP*D, and Cv*FAP*T transformants showed 1.7, 2.4, and 2.4-fold higher alka(e)ne titers, respectively (*n* = 3 biologically independent samples). **d** Relative copy numbers of the Cv*FAP* gene were quantified and showed a positive correlation with alka(e)ne titers (*n* = 3 biologically independent samples). **e** In addition, pentadecene (C15:1) derived from palmitoleoyl-CoA was detected in these strains but not the parental strains (observed from at least three biologically independent samples). **f** Alka(e)ne production of Cv*FAP*, Cv*FAP*D, and Cv*FAP*T transformants from YLjbl-20 was improved as well, but not as much as the transformants from YLjbl-19 (*n* = 3 biologically independent samples). Fermentations in (**b**) were performed in 50 mL glass conical shake flasks with a working volume of 13 mL and an initial OD_600_ of 0.1. Samples were collected after 3 days of cultivation in the dark followed by 3 days in blue light generated by light source 1 (Supplementary Fig. 16). Other fermentations were performed in 12-well microplates with a working volume of 2 mL and an initial OD_600_ of 0.5. Samples were collected after 2 days of cultivation in the dark at 30 °C followed by 1 day of cultivation in blue light generated by light source 2 at 25 °C (Supplementary Fig. 16). Fermentation medium was composed of 20 g/L glucose, 6.9 g/L yeast nitrogen base (without amino acids), and 1 g/L yeast extract. Data represent mean value ± SD. Source data underlying Fig. 3b–d, f are provided as a Source Data file.

We next sought to engineer the strain further by knocking out enzymes that are related to alka(e)ne assimilation (Fig. 3a). Though the active FAA1 was expected to be beneficial for alka(e)ne production according to the finding that acyl-CoA was the preferred substrate for *Cv*FAP, deletion of *FAA1* did not decrease alka(e)ne titers (Fig. 3b). It may be because that no pathways for FFA formation were incorporated in these strains such that the deletion of *FAA1* did not decrease the acyl-CoA pool. Surprisingly, the alka(e)ne titers increased by the double deletion of *FAA1* and *ALK2* (Fig. 3b), which might be attributed to the synergistic effect between FAA1 and ALK2 based on the fact that FAA1 is relevant to the assimilation of n-alkanes^19^. Furthermore, we found that the total lipid titer of a strain overexpressing FAA1 was significantly higher than its parental strain with Δ*FAA1* (Supplementary Fig. 6), whereas the alka(e)ne titer decreased by more than 3-fold. This result indicates that Δ*FAA1* plays a significant role in regulating the distribution of acyl-CoA to either alka(e)nes or triglycerides. Overall, Δ*FAA1* mainly played two roles, acting with Δ*ALK2* synergistically and regulating distribution of acyl-CoA between alka(e)nes and lipids.

Overexpression of Cv*FAP* in strains with double deletions of *FAA1* and *ALK2* and triple deletions of *FAA1*, *ALK1*, and *ALK2* enhanced alka(e)ne synthesis more profoundly (Fig. 3b). These results further corroborate the idea that the bottleneck for alka(e)ne production in *Y. lipolytica* is not the supply of precursors but rather the decarboxylation step. As such, we constructed plasmids carrying 2 (Cv*FAP*D) and 3 (Cv*FAP*T) copies of the Cv*FAP* gene expression cassette (Supplementary Fig. 7a) and transformed them into different strains. Compared with the parental strain YLjbl-19, Cv*FAP*, Cv*FAP*D, and Cv*FAP*T transformants showed 1.7, 2.4, and 2.4-fold higher alka(e)ne titers, respectively (Fig. 3c). Relative copy numbers of the Cv*FAP* gene in genome and mRNA were quantified and showed a positive correlation with alka(e)ne titers (Fig. 3d, Supplementary Fig. 7b). In addition, pentadecene (C15:1) derived from palmitoleoyl-CoA was detected in these strains but not the parental strains (Fig. 3e), possibly attributed to the enhanced decarboxylation efficiency. Alka(e)ne production of Cv*FAP*, Cv*FAP*D, and Cv*FAP*T transformants from YLjbl-20 was improved as well, but not as much as the transformants from YLjbl-19 (Fig. 3f). Overall, by increasing the Cv*FAP* copy number coupled with the deletion of *FAA1* and *ALK2*, we were able to obtain a strain capable of producing 35 mg/L alka(e)nes from 20 g/L glucose in a batch fermentation carried out in 12-well plates.

### Optimization of fermentation conditions for maximizing acyl-CoA to alka(e)ne flux

Strain YLjbl-26 was selected for further process optimization as it was most promising for alka(e)ne production. Our previous results (Fig. 2h) suggested implementing fermentation conditions that enhance acyl-CoA synthesis as well as its conversion to alka(e)nes rather than triglycerides. The optimization should also attempt to maximize cell biomass but not to the detriment of alka(e)ne synthesis. To this end, the following important process parameters were considered: (a) light intensity, which influences the efficiency of decarboxylation, (b) C/N ratio and glucose concentration, which impact the extent of acyl-CoA synthesis flux, and (c) switching time from dark to light conditions, which affects the transition from biomass accumulation to alka(e)ne synthesis.

In previous studies, the yield of pentadecane from decarboxylation of palmitic acid in vitro increased linearly with light intensity^11^. However, it is unclear whether increasing light intensity would have the same effect in vivo due to possible issues with light penetration in a microbial culture. We, therefore, investigated two light sources with similar emission spectra (Supplementary Fig. 8a) but different intensities (Supplementary Fig. 8b). Between the two, light source 2 with higher intensity enhanced alka(e)ne production regardless of cell density (Supplementary Fig. 8c, d), suggesting that light penetration in the tested culture was not an issue. In line with a previous report^14^, we also observed that a much higher proportion of C17:1, C17:2, C15:0, and C15:1 was produced under higher light intensity (Supplementary Fig. 8c), indicating that the enzymatic specificity of *Cv*FAP was light intensity dependent. The maximum titer reached 110 mg/L using the higher intensity light and a favorable C/N ratio. The optimal C/N ratio was determined to be approximately 43, which perfectly balances the trade-off between more resources allocated to alka(e)ne synthesis versus more biomass to house the alka(e)nes (Supplementary Fig. 8d). Additionally, since there is little information available regarding the timing of light activation, we studied extensively how the onset of light exposure affects the culture (Supplementary Fig. 9). Cultivation without an initial dark phase showed the slowest growth and glucose consumption as well as the lowest alka(e)ne production (Supplementary Fig. 9a). Thus, we allowed the cells to grow and accumulate biomass under dark conditions prior to activating alka(e)ne synthesis. It was determined that cultivating the cells in the dark for 2 days followed by 1 day in the light was the optimal condition (113 mg/L), with other light patterns yielding poorer performance (Supplementary Fig. 9).

**Fig. 4:**
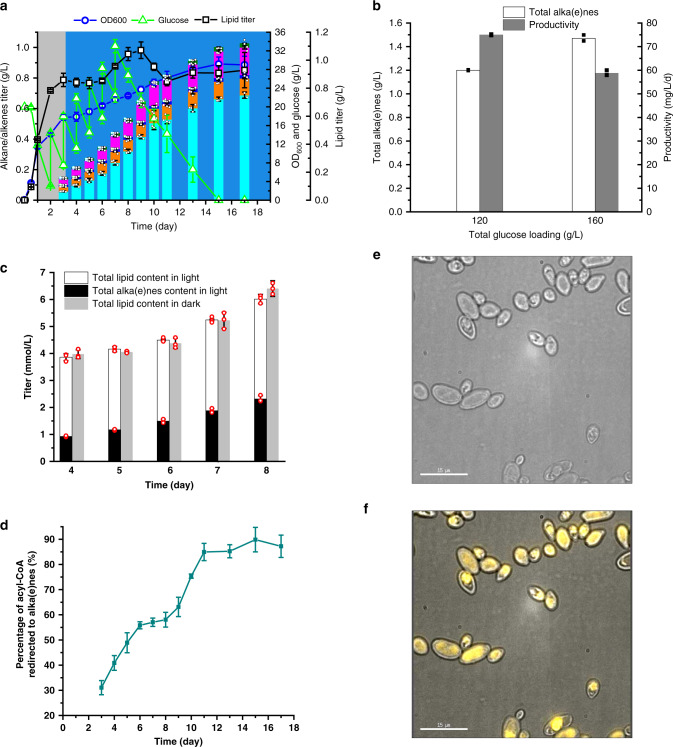
Redirection of acyl-CoA to form alka(e)nes during fed-batch fermentation process. **a** Production of alka(e)nes by YLjbl-26 in fed-batch fermentations. Gray-colored regions indicate cultivation in the dark while blue-colored regions indicate cultivation in blue light (*n* = 3 or 4 biologically independent samples). **b** Alka(e)ne titers and productivities from different glucose loadings with the same feeding strategy as (**a**) (*n* = 2 biologically independent samples). **c** The amount of lipids in cells cultured in dark conditions matched the sum of lipids and alka(e)nes in cells cultured in light conditions. Both fermentations were carried out following the same feeding strategy as shown in (**a**). **d** We further developed a method (Supplementary Note 2) to determine the fraction of the redirected flux. This fraction increased from 31% to 85% as fermentation progressed from day 3 to 11, asymptotically reaching a maximum of 89%. **e** Brightfield microscopic images of cells with alka(e)ne titers of 1.03 g/L, equivalent to 89% of neutral lipids. **f** Fluorescence images of cells (yellow), merged with the brightfield images. Staining our alka(e)ne-producing strain reveals that most cells were filled with intracellular lipids and alka(e)nes with no evidence of extracellular fluorescence. Images were taken by a DeltaVision2-TIRF Microscope. Cells were stained by Nile red. Fermentations were performed in 50 mL glass conical shake flasks with a working volume of 13 mL and an initial OD_600_ of 0.1. Blue light was generated by light source 2 (Supplementary Fig. 16). Data represent mean value ± SD, *n* = 3 or *n* = 4 biologically independent samples. Source data underlying Fig. 4a–d are provided as a Source Data file.

High initial glucose concentrations inhibit cell growth, substrate uptake, and lipid synthesis (Supplementary Fig. 10), in agreement with a previous report^12^. We thus investigated a fed-batch fermentation mode that allowed us to maintain relatively low glucose levels (Fig. 4a). Alka(e)ne titers increased by around 75 mg/L/day in the first 10 days where sufficient glucose was present. The production rate then slowed down afterward to about 50 mg/L/d, yielding a final alka(e)ne titer of 1.03 g/L after 17 days. We examined the effect of C/N ratio in the feeding media and found that feeding without nitrogen produced less alka(e)nes than those with nitrogen supplementation (Supplementary Fig. 11). This suggested that the C/N ratios of both the initial and feeding media should be well controlled. Following the same feeding strategy, alka(e)ne titers of 1.20 and 1.47 g/L were obtained from 120 and 160 g/L glucose, respectively (Fig. 4b), which are the highest reported so far in yeast.

Our data showed that the amount of lipids in cells cultured in dark conditions matched the sum of lipids and alka(e)nes in cells cultured in light conditions (Fig. 4c), which suggests redirection of acyl-CoAs from lipids to alka(e)nes. Accordingly, we further developed a method (Supplementary Note 2) to determine the fraction of the redirected flux. This fraction increased from 31% to 85% as fermentation progressed from day 3 to 11, asymptotically reaching a maximum of 89% (Fig. 4d, Supplementary Fig. 12), providing evidence that the majority of acyl-CoA was rewired towards alka(e)ne synthesis during the light period.

We also examined the subcellular localization of the formed alka(e)nes. We first confirmed that alka(e)nes can fluoresce when emulsified in water with Tween 80 and stained with Nile red (Supplementary Fig. 13). Staining our alka(e)ne-producing strain reveals that most cells were filled with intracellular lipids and alka(e)nes with no evidence of extracellular fluorescence (Fig. 4e, f). Assuming that with further optimizations, the intracellular accumulation of alka(e)nes can reach the levels reported for lipids, this product sequestration could offer unique advantages in its downstream separation from the other fermentation components.

Finally, alka(e)ne toxicity was investigated for YLjbl-26. The addition of 1.6 g/L alka(e)nes in the fermentation medium did not affect cell growth (Supplementary Fig. 14), suggesting no toxicity from exogenous sources. Cell viability of YLjbl-26 was compared between a culture performed in dark conditions and a culture performed in normal alka(e)ne-producing conditions (86.18 mg/L titer). Using trypan blue staining, no dead cells were found in either culture (Supplementary Fig. 15), indicating that intracellular alka(e)nes did not pose observable toxic effects to the host cell. There was also no interference to acyl chain synthesis from the presence of intracellular alka(e)nes, as indicated in Fig. 4c. Overall, it can be concluded that the inhibitory effects of sequestered alka(e)nes to *Y. lipolytica* are negligible.

### Production of alka(e)nes from CO_2_-derived substrates

**Fig. 5:**
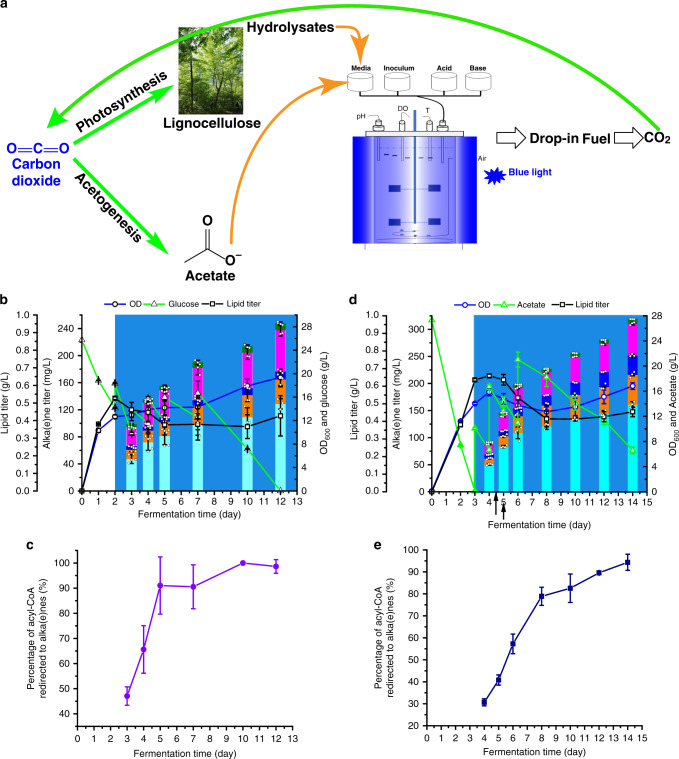
Alka(e)ne production from two CO_2_-neutral substrates. **a** Schematic diagram to show how the strain and process developed in this work could advance the net-zero GHG emissions by using CO_2_-neutral feedstocks other than corn or sugar canes-sourced glucose. **b** Fermentation time course of alka(e)ne production from wheat straw hydrolysates. **c** The percentage of acyl-CoAs that were redirected to form alka(e)nes during wheat straw hydrolysate fermentation. **d** Fermentation time course of alka(e)ne production from sodium acetate. **e** The percentage of acyl-CoAs that were redirected to form alka(e)nes during acetate fermentation. Fermentations were performed in 50 mL glass conical shake flasks with a working volume of 13 mL and an initial OD_600_ of 0.1. Blue light was generated by light source 2 (Supplementary Fig. 16). Data represent mean value ± SD, *n* = 3 biologically independent samples. J. Li created the tree image in (**a**). Source data underlying Fig. 5b–d are provided as a Source Data file.

The products made with our strains are suitable for use as jet fuel (C15–C19)^26^ and diesel (C9–C23)^27^, both of which are important for the aviation industry and heavy vehicle transport. However, to achieve net-zero GHG emissions in these sectors without potential competition with food supply, CO_2_-neutral feedstocks other than corn or sugar cane-sourced glucose must be investigated. We thus examined the compatibility of our strain with two other notable feedstocks, wheat straw hydrolysate and acetate, which are primary products of CO_2_ fixation by plants and acetogens^28^, respectively (Fig. 5a).

Fermentation of wheat straw enzymatic hydrolysate was carried out following the developed fed-batch fermentation (Fig. 5b). Compared to the fermentation of glucose, alka(e)ne titers increased at a slower rate due to lower glucose concentrations and the possible presence of inhibitors^29^. The final titer and yield after 12 days of fermentation reached 242.04 mg/L and 4.66 mg/g (glucose+yeast extract), respectively, with a productivity of 24.20 mg/L/d. The percentage of acyl-CoAs redirected to alka(e)nes increased with fermentation time and reached more than 95% after 10 days of fermentation (Fig. 5c).

Conversion of acetate to alka(e)nes was also very promising. Due to the slower growth in the acetate medium, cells were cultured in the dark phase for 3 days prior to light exposure (Fig. 5d). The uptake of acetate was slower than that of glucose. Alka(e)ne production ceased after 14 days reaching a final titer of 318.70 mg/L, equivalent to a yield of 5.72 mg/g (acetic acid+yeast extract) and productivity of 28.98 mg/L/d. Acetate thus outperforms wheat straw hydrolysates. The maximum fraction of acyl-CoAs that was diverted to alka(e)nes reached 94% at the end of the fermentation (Fig. 5e).

## Discussion

Sorigué et al.^11^ discovered the fatty acid photodecarboxylase *Cv*FAP in response to blue light, characterized its blue light dependence, and elucidated its crystal structure. The enzyme was later expressed in *Y. lipolytica* strains with different genotypes for alka(e)ne production^14^. However, the detailed effects of each step along the metabolic pathway for alka(e)ne synthesis were not thoroughly studied. Our work elucidated the necessary steps for in vivo alka(e)ne production and identified the preferred substrate for the *Cv*FAP, resulting in a modified metabolic pathway that enabled the redirection of large proportion of acyl-CoA (89%) to alka(e)nes. Driven by the deeper understanding, our work enhanced the alka(e)ne titers substantially compared with previous study^14^. For example, expression of Cv*FAP* in an FFA-overproducing strain (approx. 2 g/L intracellular and 2.5 g/L extracellular FFAs^20^) produced 58.7 mg/L alka(e)nes using optimized fed-batch strategy^14^. By comparison, our highest titer from fed-batch fermentation (1.47 g/L) was 25-time more than that obtained from the previous study^14^. The main reason for the significant enhancement was the finding that acyl-CoA was the substrate for *Cv*FAP and the modified metabolic pathway thereof.

The proposed pathway (Fig. 2h) suggests that FFA is not necessary for alka(e)ne production, thus reducing the accumulation of this toxic intermediate in the cell^16,20^. This also indicates that the expression of thioesterases or lipases is not required. Additionally, with a large pool of acyl-CoAs naturally present in *Y. lipolytica*, the employed debottlenecking strategy by simply increasing the Cv*FAP* copy number can be very effective and requires less pathway engineering, thus reducing the burden of expressing multiple genes and balancing other pathways.

Furthermore, there are additional advantages of the discovered pathway. Previous efforts in microbial alka(e)ne production focused on the cyanobacterial pathway where acyl-ACP/acyl-CoA needs to be reduced to its corresponding aldehyde prior to decarbonylation^7,30^. The biological reduction step requires reducing equivalents in the form of NAD(P)H^7^. Similarly, the OleT_JE_ P450 catalyzed decarboxylation reaction to form alkenes directly from FFAs also requires NAD(P)H as a cofactor^31,32^. By contrast, the engineered strains in our work use a photo-driven decarboxylase that relies on extracellular photons from blue light to produce alka(e)nes, bypassing the NAD(P)H requirement. This allows the decoupling of alka(e)ne production from these cofactors, conserving NAD(P)H for biomass and acyl chain synthesis that are oftentimes limited by NAD(P)H availability in *Y. lipolytica*^33^. It is also noteworthy that alka(e)nes with specific composition may be tailored by altering the light intensity (Supplementary Fig. 8c) or engineering *Cv*FAP^14^.

The endogenously formed alka(e)nes exhibited neglectable toxicity and were sequestered intracellularly (Fig. 4e, f), which may be facilitated by the intrinsic ability of *Y. lipolytica* to form lipid bodies^34^. Sequestering products inside cells offers simpler downstream processing for product recovery as there is no need to extract and separate products from the fermentation broth, significantly lowering the production cost. Overall, the benefits of alka(e)ne sequestration and the ability of *Y. lipolytica* to naturally drive more carbon flux to acyl-CoAs make it a preferred organism for alka(e)ne production.

With an initial glucose loading of 80 g/L, 1.03 g/L alka(e)nes and 0.93 g/L lipids were produced, corresponding to a yield of approximately 24.5 mg/g glucose, which was comparable to that of a wild-type *Y. lipolytica* strain expressing β-galactosidase (23 mg/g)^24^. Though the comparable yield was achieved, we explored more of the carbon balance and found that apart from alka(e)nes and lipids, the major byproducts in the fermentation broth were determined to be 33.01 g/L pyruvate and 2.74 g/L citrate. Assuming similar yields of these products to that reported in the literature^35–37^, the measurements satisfy a closed carbon balance. The results also guide us that future works should be focused on directing the pyruvate flux to acyl-CoA to further enhance the alka(e)ne yield, which may be achieved by enhancing pyruvate dehydrogenase^38^ or introducing pyruvate dehydrogenase bypass^39^. Besides, increasing acetyl-CoA pool by feeding acetate as direct substrate^39^ following the substrate co-feeding strategy^28^ may also improve alka(e)ne titer. Overexpression of citrate shuttle consisting of ATP-citrate lyase^40^ may increase acetyl-CoA pool and thereby enhance alka(e)ne production as well.

By employing the engineered strain and the developed process, two CO_2_-derived substrates were converted to alka(e)nes effectively. Further engineering of YLjbl-26 to utilize xylose^41^ and tolerate inhibitors could increase the yield of alka(e)nes from lignocelluloses. With regards to acetate conversion, the energy efficiency of H_2_/CO_2_-to-acetate-to-lipids conversion can reach 38%^28^. Considering that 94% of acyl-CoAs were converted to alka(e)nes (Fig. 5e), we can estimate that a potential process generating alka(e)nes from H_2_/CO_2_ would have a theoretical efficiency of 35.7% if the co-feeding strategies^28^ were adopted. With further development of the conventional bioreactors equipped with internal blue light bulbs focusing on controlling the light intensity and enhancing penetration, the scale-up of either process to produce drop-in fuels with tailored composition would be possible. These fuels can be used directly in our existing infrastructure, transforming the current long-distance road transport, aviation, and shipping to a more sustainable one.

## Methods

### Strains and reagents

*Y. lipolytica* strains and plasmids used are listed in Supplementary Tables 2 and 3. KAPA HiFi DNA Polymerase with high-fidelity was purchased from KapaBiosystems. GoTaq Green Master Mix was purchased from Promega Corp and used for colony PCR. Gibson assembly, chemical competent *E. coli* cell (DH 5α), and restriction enzymes were purchased from New England Biolabs. DNA gel recovery and plasmid extraction kits were purchased from Qiagen. All heterologous genes were codon-optimized and synthesized by ThermoFisher Scientific. All primers were synthesized by MilliporeSigma. Hexane, methanol, H_2_SO_4_, NaOH, and dodecane were purchased from MilliporeSigma. Yeast extract and peptone were from Bacto. The nitrogen content of the yeast extract was 11% (w/w). Yeast nitrogen base without amino acids and ammonium sulfate (YNB-AA^-^AS^-^) was from VWR Life Science. Alkane standards were purchased from VWR with purity higher than 98%.

Wheat straw was pretreated according to the previous work^42^. Briefly, 50 g wheat straw was cut to pieces with the average length of roughly 0.5 cm and added to 500 mL of 2% NaOH aqueous solution to a final solid concentration of 10%. The mixture was subsequently subjected to 121 °C for 1 h and filtered through a 20 mesh (0.85 mm) filter to collect the solids. The solids were then washed to reach neutral pH, concentrated by pressing water out, and yielded around 140 g pretreated wheat straw with water content of approximately 75%. This mixture was directly hydrolyzed by adding 10 mL of citrate buffer (pH 5.5) and 2 mL Cellic CTec2 (Sigma-Aldrich) for 4 days at 50 °C and 250 rpm. The final glucose concentration was 83.8 g/L in the hydrolysate.

Bjour Curtain String Lights 300 LED Window Curtain Icicle Blue Light manufactured by Twinkle Star was purchased from Amazon (Light source 1). Water-Resistance IP65, 12 V Waterproof Flexible LED Strip Light, 16.4 ft/5 m Cuttable LED Light Strips, 300 Units 2835 LEDs Lighting String manufactured by Tasodin was purchased from Amazon (Light source 2). The LED power was measured with a PD300-3W photodiode sensor (Ophir Photonics, https://www.ophiropt.com/laser–measurement/laser-power-energy-meters/products/Laser-Photodiode-Sensors/Standard-Photodiode-Sensors/PD300-3W) set at the LED peak wavelength connected to a Vega power meter (Ophir Photonics, https://www.ophiropt.com/laser–measurement/laser-power-energy-meters/products/smart-displays/vega).

### Genetic manipulation

Gene fragments were either synthesized as shown in Supplementary Data 1 or amplified with primers listed in Supplementary Table 5. Gibson Assembly was used to ligate the fragments into ring form plasmids. Transformation of the resultant mixture into *E. coli* DH 5α by heat shock at 42 °C for 1 min, followed by plating cells on Petri dishes with LB agar containing corresponding antibiotics were performed. Colonies were checked with colony PCR using primers on promoter and structural gene to select positive ones. All plasmids harboring one cassette were constructed by the above-described procedures. Plasmids harboring repeated pTef-in-Cv*FAP*-XPR2t cassette were constructed by the single restriction enzyme digestion, followed by Gibson Assembly. Plasmids were extracted from the cultivated positive strains and sequenced. All plasmids used in this work are listed in Supplementary Table 2.

All plasmids were linearized by restriction enzyme NotI or AseI before transformation into auxotrophic *Y. lipolytica* (*URA3*^-^). The transformation medium was composed of 2.25 mL of 50% PEG 3350 in water, 0.125 mL 2 M lithium acetate, and 0.125 mL 2 M DTT in water. Transformation medium of 100 μL, 5 μL of single-strand DNA (10 g/L), and plasmid DNA of interest (1 μg) were mixed together with the auxotrophic parent strain, vortexed for 15 s, and placed in 28 °C for 30 min, followed by incubating at 39 °C for 30 min. The resultant mixture was spread on YNB-Uracil^-^ agar plate (see below for the composition) and allowed to be in 30 °C incubator for 2–3 days. Recombinants were checked by colony PCR and the positive ones were selected for the first-round screening fermentation.

Gene knockouts were performed according to the developed CRISPR method^43^. Briefly, gRNA sequences were designed by Benchling with NGG as PAM. Homologous arms with 100 bps composed of 50 bps from the upstream and 50 bps from downstream were used to delete the part in between. The positive knockouts were examined by PCR with genomic DNA as templates. All primers, homologous arms, and gRNA sequences related to gene knockouts are listed in Supplementary Table 4.

After each transformation or knockout, colony PCR-positive *Y. lipolytica* strains were further screened by microplate fermentation. Briefly, 12 positive strains were inoculated to 12-well microplate containing 2 mL YEM medium in each well and cultivated in dark at 30 °C, 225 rpm for 2 days, followed by cultivation in blue light at 25 °C, 150 rpm for 1 day prior to alka(e)ne analyses. Strains with the highest alka(e)ne titer were selected for further studies.

Counterselection against 5 -Fluoroorotic acid (5-FOA) was performed to recover *URA3* marker. Briefly, gene fragment of 1000 bp containing both upstream and downstream of *URA3* was transformed into the *Y. lipolytica* strains of interest, followed by spreading on yeast nitrogen base medium (YNB-FOA) containing 1 mg/mL 5-FOA and 5 mg/mL uracil. The colonies that grew over 3 days were transferred onto an uracil-minus plate and a corresponding YPD plate to confirm the auxotroph.

### Media and conditions

Luria–Bertani (LB) medium containing 10 g/L peptone (Bacto), 5 g/L yeast extract (Bacto), 10 g/L NaCl (Sigma), and corresponding antibiotics (carbenicillin 100 μg/mL, kanamycin 50 μg/mL, or chloramphenicol 34 μg/mL) was used to culture *E. coli* harboring plasmid. Culturing tubes with the inoculated medium was placed on a rotatory drum at 37 °C for around 15 h for plasmid proliferation.

Different solid media were prepared as follows. YNB-Uracil^-^ medium composed of 1.7 g/L YNB-AA^-^SA^-^, 20 g/L glucose (Sigma), 5 g/L ammonium sulfate, 15 g/L agar (Bacto), with an appropriate supplement of 0.77 g/L complete supplement mixture minus uracil (Sunrise science products) was used for selecting transformed *Y. lipolytica* strains. YNB-FOA medium containing 1.7 g/L YNB-AA^-^SA^-^, 5 g/L ammonium sulfate, 20 g/L glucose, 0.77 g/L complete supplement mixture and 1 g/L 5-fluoroorotic acid (Zymo Research) was used to counterselect the *URA3*-disrupted strains. YPD medium containing 10 g/L yeast extract, 20 g/L peptone, 20 g/L glucose was used to culture auxotrophic strains.

For alka(e)ne fermentation before process optimization, YEM medium composed of 1 g/L yeast extract, 6.9 g/L YNB-AA^-^SA^-^, and 20 g/L glucose was used unless stated otherwise.

### Batch fermentation

Batch fermentations for alka(e)ne production were performed in either 50 mL conical glass flasks or Falcon polystyrene 12 well microplates for either 3 days or 2 days in dark followed by 3, 2, or 1 day in blue light. The temperature was controlled at 30 °C in dark while 25 °C in light. The light sources with a maximum peak wavelength of 454 and 458 nm (Supplementary Fig. 8a) were applied during light cultivation phase. The working volume for 50 mL conical glass flasks and 12 well plates was 13 mL and 2 mL, respectively with inoculum of the initial OD_600_ of 0.1 and 0.5, respectively. The self-made instrument for cultivation in blue light is shown in Supplementary Fig. 16.

### Batch fermentations with supplementation of palmitic acid-d_31_

Fermentations were carried out by using strains YLjbl-2 and YLjbl-2-Δ*FAA1*. Medium containing 2 g/L yeast extract, 6.9 g/L YNB-AA^-^SA^-^, and 20 g/L glucose was used. Palmitic acid-d_31_ (dissolved in absolute ethanol) with a final concentration of 5 g/L was added before switching to blue light cultivation. In control experiments, the same amount of absolute ethanol was added.

### Fed-batch fermentation with glucose

Medium containing 2 g/L yeast extract, 6.9 g/L YNB-AA^-^SA^-^, and 20 g/L glucose was used for cell growth for 2 days in dark. Thereafter, glucose or glucose with different amounts of yeast extract composed of 200 g/L glucose and 5, 10, 15 g/L yeast extract, equivalent to a C/N ratio of 170, 85, and 57 was fed every 24 h until the total glucose reached 80, 120, and 160 g/L. From day 2 to day 7, the feeding medium was supplemented to increase the glucose concentration by 10 g/L each day. pH was not controlled. All fed-batch fermentations were carried out in 50 mL conical flasks with a working volume of 13 mL for 2 days in dark followed by culture in blue light generated by light source 2 at 25 °C (Supplementary Fig. 16). The initial OD_600_ was 0.1 and feeding was conducted when the increase in glucose/acetate could be observed in Figs. 4 and 5.

### Fed-batch fermentation with acetate

For fed-batch fermentation with acetate, medium containing 2 g/L yeast extract, 6.9 g/L YNB-AA^-^SA^-^, and 27.3 g/L sodium acetate was used for cell growth for 3 days in dark. Acetic acid was added at 4.5 days and 5 days to adjust pH to 6.8. Sodium acetate (273 g/L with 5 g/L yeast extract) was used as a feeding medium. The acetic acid added was 2.31 g/L and in total 57.3 g/L acetic acid equivalence was added. Yeast extract of 2.75 g/L was added during the whole fermentation process. For stabilizing pH, 5 mM PBS (pH 6.0) was used at the initial stage and HCl (6 M) was used to control pH around 6.8 every 24 h.

### Fed-batch fermentation with wheat straw hydrolysate

For fed-batch fermentation with wheat straw hydrolysate, medium containing 1 g/L yeast extract (optimized), 6.9 g/L YNB-AA^-^SA^-^, and wheat straw hydrolysate with final glucose of 25.74 g/L was used for cell growth for 2 days in dark. The hydrolysate with a glucose concentration of 83.8 g/L without yeast extract supplement was used as feeding medium for 6 times. The total glucose and yeast extract added were 50.89 g/L and 1 g/L yeast extract, respectively, during the whole fermentation process without controlling the pH.

### Analytical methods for metabolites

OD_600_ was measured by a spectrophotometer against corresponding blanks. Dilutions were carried out to make sure the OD_600_ value ranging from 0.1 to 0.9.

Glucose and acetic acid in the supernatant of cultures were analyzed by high-performance liquid chromatography (HPLC, Agilent Technologies, 1260 Infinity) equipped with a BioRad HPX-87H column (BioRad) and a refractive index detector (Agilent Technologies, 1260 Infinity). External standard curves using corresponding compounds with analytical grades were prepared for quantification purposes. Agilent OpenLab Software 7890B was used to collect and analyze data.

For alkane/alkene quantification, cell pellets from 0.05 to 1 mL cell culture depending on the content were collected by centrifuge at 18,406.75 × *g* for 5 min. Sodium hydroxide-methanol solution (0.5 mol/L) of 0.5 mL was mixed with cell pellets, followed by adding internal standards, glyceryl triheptadecanoate (Sigma, 2 g/L) and dodecane (Sigma, 37.5 mg/L) in hexane. The mixture was sonicated for 15 min and vortexed (Vortex-Genie 2, Scientific Industries) for 1 h at room temperature (24 ± 2 °C) prior to being acidified by adding 60 μL condensed H_2_SO_4_ (Sigma) with caution. Hexane (0.5 mL) was added and vortexed for 30 min to extract fatty acid methyl esters and alkanes/alkenes. External standard curves were prepared by running the same procedure using corresponding standard compounds of analytical grade. The upper phase after centrifugation was analyzed by GC-MS (Agilent Technologies, 7890B Series GC and a 5977B MS) or GC-FID (Agilent 7890B). For GC-MS, 3 μL of extract in hexane was injected without split into an HP-5MS UI column (Agilent Technologies). Whereas for GC-FID, 1 μL of extract in hexane was injected with the split ratio of 2:1 into an HP-INNOWAX column (Agilent Technologies). For GC-FID, the inlet and FID temperature was held at 260 °C using the following temperature program: initial 2 min at 40 °C, then ramped to 200 °C at a rate of 20 °C/min, held for 2 min, and ramped to 250 °C at a rate of 40 °C/min, and held for 5 min. For GC-MS, the inlet, transfer tube, and MS source temperatures were set at 320, 300, and 280 °C, respectively. The MS was operated in scan mode from 100 to 450 *m/z*. The temperature program was: initial 2 min at 40 °C, then ramped to 270 °C at a rate of 12 °C/min, and ramped to 310 °C at a rate of 6 °C/min, and held for 10 min. The standard curves of different alka(e)nes were shown in Supplementary Fig. 17. Agilent OpenLab Software 7890B was used to collect data.

For FFA quantification, 0.5 mL culture was mixed with 80 μL glass beads, 150 μL methanol, and 150 μL hexane containing pentadecanoic acid as internal standard. The mixture was rigorously vortexed for 2 h, followed by centrifugation to facilitate phase separation. The upper hexane phase was taken for GC-MS analysis. The inlet, transfer tube, and MS source temperatures were set at 320, 300, and 280 °C, respectively. The MS was operated in scan mode from 150 to 500 *m/z*. The temperature program was: initial at 100 °C, then ramped to 270 °C at a rate of 12 °C/min, and ramped to 310 °C at a rate of 6 °C/min, and held for 10 min. Helium was used as a carrier gas.

### Substrate docking and structural analysis

Crystal structure images were produced by using the UCSF Chimera package from the Computer Graphics Laboratory, University of California, San Francisco (supported by NIH P41 RR‐01081)^44^. Substrate docking was performed by using AutoDock Vina 1.1.2, with implementation through Chimera 1.13.1rc^45^. Protein structure (with fatty acid substrate and FAD deleted) and fatty acyl-CoA substrate were prepared by using the DockPrep tool of Chimera. Analysis of the resulting docking modes was performed through the ViewDock tool, which listed 10 docking modes according to their energy scores. The best docking model was decided by a combination of criteria: visual inspection (correct orientation of the fatty acid and nucleotide ends of acyl-CoA, match of the nucleotide binding site) and energy scores.

### Quantitative real-time PCR and reverse transcription–qPCR analyses

For quantitative real-time PCR, total genomic DNA extracted from each individual strains of interest by using the Phenol-Chloroform protocol was used as template. For reverse transcription–qPCR, mRNA extracted by MasterPure^TM^ Yeast RNA purification kit (Lucigen, Wisconsin, USA) was used as template. PCRs were performed using iScript^TM^ one-step RT-PCR kit with SYBR^®^ green (Bio-Rad) with the designed primers of Cv*FAP* and internal reference *ACT* (Supplementary Table 5) on an iCycler (Bio-Rad), according to the manufacturer’s instructions. Relative gene expression was performed using the comparative 2^-∆∆Ct^ or 2^-∆Ct^ method.

### *Cv*FAP protein expression and purification

The construct consisting of the Cv*FAP* gene with an optimized codon for *Y. lipolytica* and a sequentially 6×His tag on 3′-terminus were cloned into a pET28 vector backbone using Gibson Assembly. The plasmid was then transformed in *E. coli* DH5α and the positive colonies were identified by colony-PCR. Three plasmids were extracted and sequenced to select the correct one for protein expression.

The selected plasmid was transformed in *E. coli* BL21 (DE3) (purchased from NEB). The recombinant was precultured in 5 mL LB medium containing 50 μg/L kanamycin overnight. The preculture was then inoculated to 500 mL medium with the same composition as the preculturing medium. IPTG was added with the final concentration of 0.5 mM when the OD_600_ reached 0.8–0.9. The culture was then moved to an incubator at 16 °C and 160 rpm for 20 h. Cells were harvested by centrifugation at 4 °C and the cell pellet was washed with Tris-HCl buffer (50 mM, pH 8.5) once.

The cell pellet was resuspended by NPI-10 for cell disruption. The suspended cells were disrupted by a sonic disruptor on ice for 30 min. Protein purification was carried out according to the protocol (https://www.mn-net.com/Portals/8/attachments/Redakteure_Bio/Protocols/Protino/UM_Protino96NTA.pdf). The resultant protein fractions were examined by SDS-PAGE as shown in Supplementary Fig. 18. Fractions from NPI-125 and NPI-250 were combined for following buffer change. Buffer change was performed by washing NPI-125 and NPI-250 fractions through 10 kD cut-off using Tris-HCl buffer (50 mM, pH 8.5) for 3 times. The obtained protein was used for in vitro photodecarboxylation experiments.

### In vitro photocatalytic reactions

The reactions using stearoyl-CoA lithium salt (Sigma) and stearic acid lithium salt as substrates were performed at 25 °C in a total volume of 100 μL. Different volumes of substrates in DMSO (0.97 mM), a fixed amount of purified enzyme (20 μL, 3.30 mg/mL), pure DMSO, and Tris-HCl buffer (50 mM, pH 8.5) were added in transparent glass vials (2 mL) with final DMSO of 30 vol%. The vials were screw-capped and placed in the shaker with blue light for 20 h at 150 rpm. The product was extracted with ethyl acetate and analyzed by the early-mentioned GC-FID method.

### Cell viability measurements

To measure cell viability, Trypan blue (Sigma-Aldrich, 0.4% in water) was added directly to aliquots of undiluted cultures with a final concentration of 0.2% and visualized immediately at 1000× magnification on a ZEISS Axioskop (West Germany) by bright field microscope. Images were recorded using a Nikon color camera.

### Staining measurements of alka(e)nes and cells containing alka(e)nes

An artificial mixture of 10 mg of alka(e)nes containing C17:0, C17:1 (terminal double bond), and C15:0 was mixed with 0.5 mL water and 100 μL Tween 80. The mixture was vigorously vortexed at 1000 rpm for 5 min before being stained by Nile red (Ethanol solution, 0.1 μg/ml). Images were taken by using a DeltaVision2-TIRF Microscope.

### Reporting summary

Further information on research design is available in the Nature Research Reporting Summary linked to this article.

## Supplementary information

Supplementary Information

Description of Additional Supplementary Files

Supplementary Data 1

Reporting Summary

## Data Availability

The data supporting the findings of this study are available within the paper and its supplementary information files. A reporting summary for this article is available as a supplementary information file. The datasets and materials generated and analyzed during the current study are available from the corresponding author upon request. Structure of fatty acid photodecarboxylase in complex with FAD and palmitic acid is available from Protein Data Base with ID 5NCC (DOI: 10.2210/pdb5NCC/pdb) [https://www.rcsb.org/structure/5NCC].  Source data are provided with this paper.

## Source data

Source Data
