# Supplementary material for: Synthesis of high-titer alka(e)nes in Yarrowia lipolytica is enabled by a discovered mechanism
Source: Nat Commun. 2020 Dec 3;11:6198. doi: 10.1038/s41467-020-19995-0 (PMC7713262; doi:10.1038/s41467-020-19995-0)
Supplement: Supplementary file 1 — Supplementary Information [file 41467_2020_19995_MOESM1_ESM.pdf]

Synthesis of high-titer alka(e)nes in *Yarrowia lipolytica* is enabled by a discovered mechanism

Li *et al.*

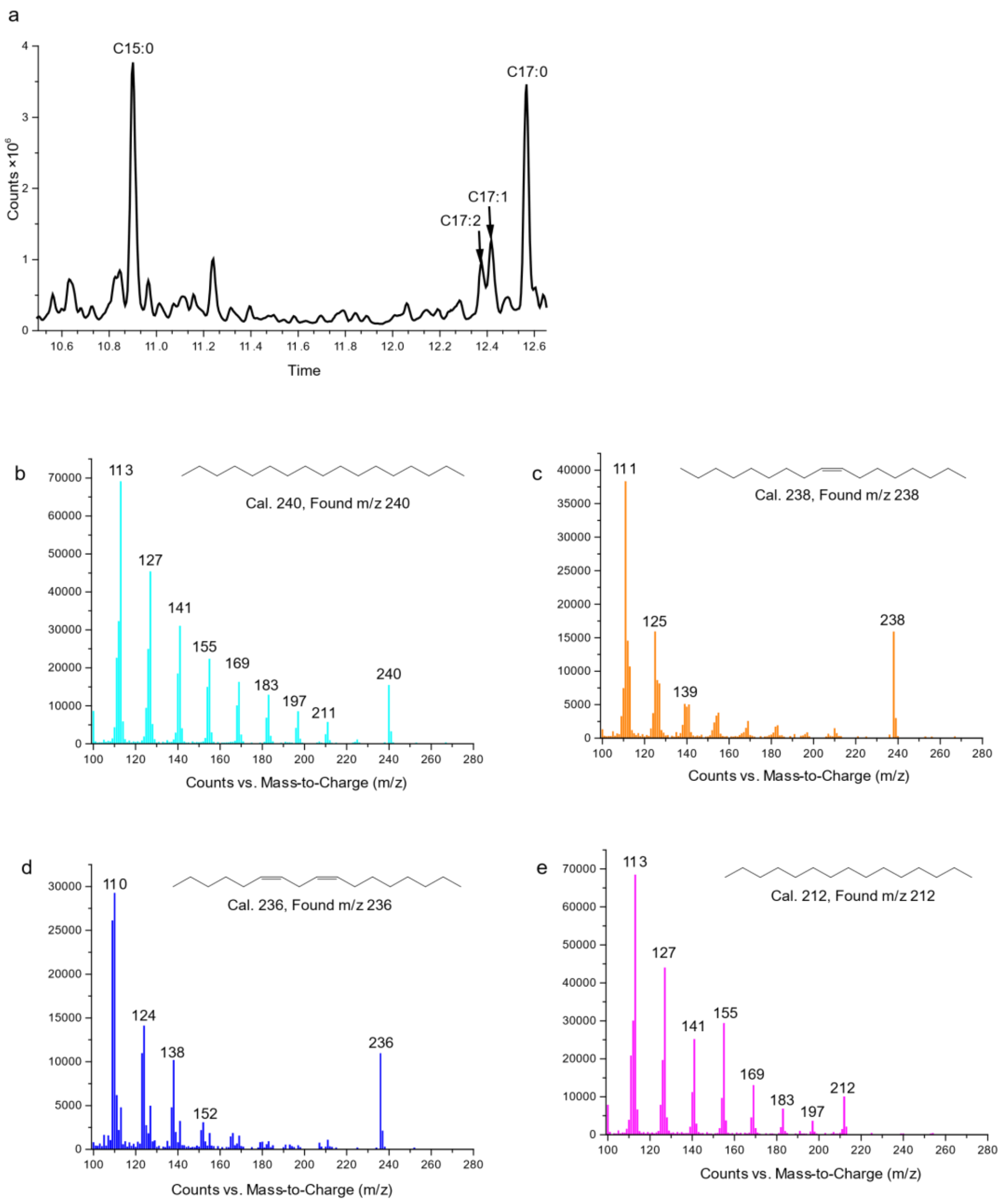

Supplementary Figure 1. GC-MS chromatogram and mass spectra of each alka(e)ne components extracted from YLjbl-2 after 3 days culture in dark followed by 3 days in blue light generated by light source 1. (a): chromatogram of formed alka(e)nes. Unsaturated compounds were eluted before the saturated ones. (b), (c), (d) and (e): mass spectra of heptadecane, 8-heptadecene, 6,9-heptadecene, and pentadecane. The molecular weights calculated are exactly the same as the found ones.

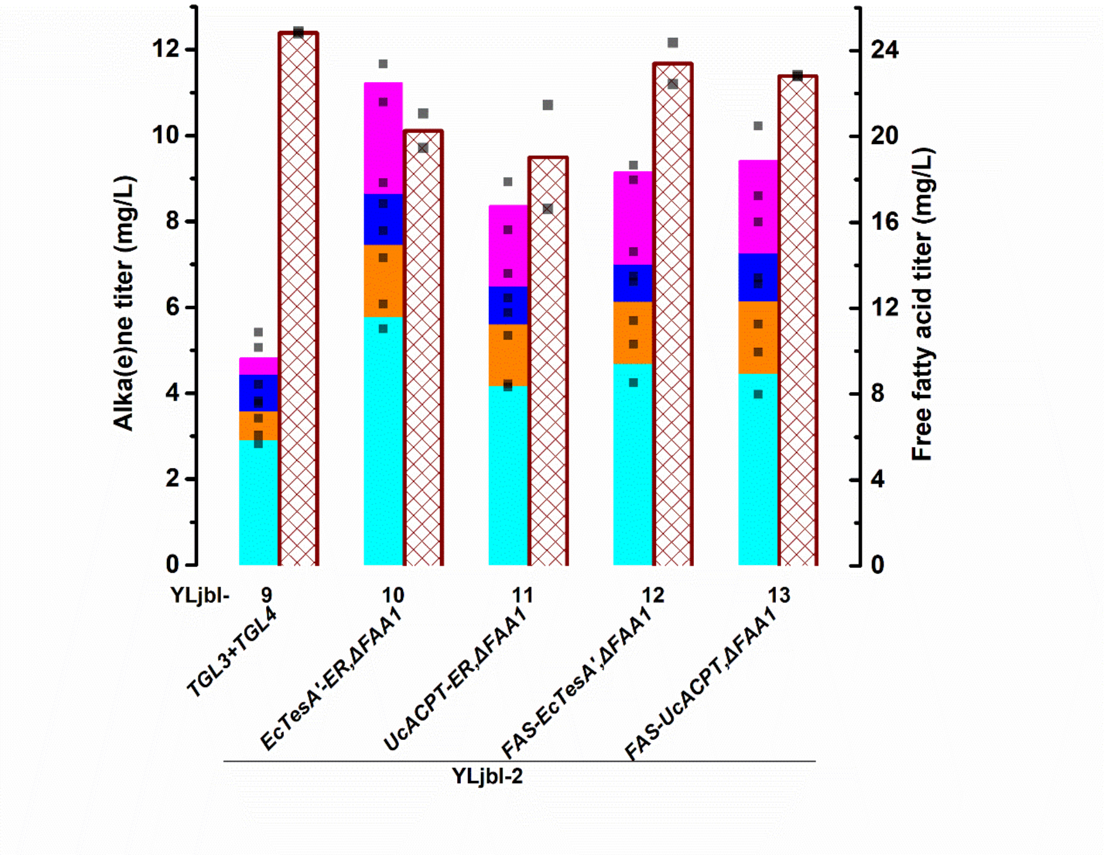

Supplementary Figure 2. Expression of thioesterases and lipases in YLjbl-2 and YLjbl-2-Δ*FAA1* enhanced free fatty acid production but decreased alka(e)ne titer. Fermentations were performed in 50 mL conical shake flasks with working volume of 13 mL and an initial OD_{600} of 0.1 for 3 days in the dark followed by 3 days in blue light generated by light source 1 (Supplementary Figure 16). Fermentation medium was composed of 20 g/L glucose, 6.9 g/L yeast nitrogen base (without amino acids), and 1 g/L yeast extract. Data represent mean value ± SD. Abbreviations: ER represents tarteting on endoplasmic reticulum. FAS-*Ec*TesA': Fusion protein of fatty acid synthase and *Ec*TesA'. TGL3 and TGL4, intracellular lipases from *Y. lipolytica*; FAA1, acyl-CoA synthase. Data represent mean value ± SD, n=2 biologically independent samples. Source data are provided as a Source Data file.

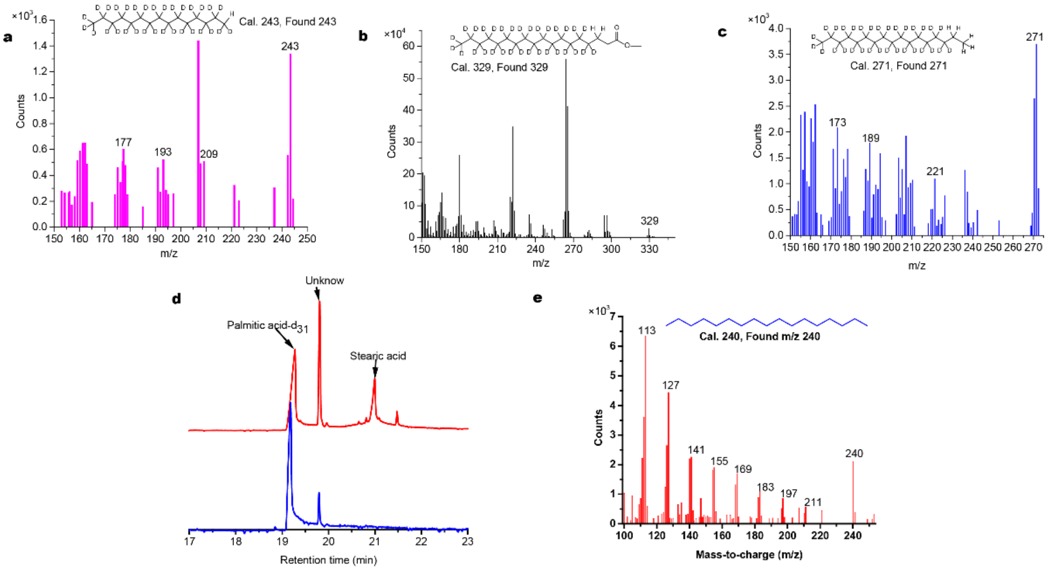

Supplementary Figure 3. Mass spectra of (a): pentadecane-d_{31}, (b): stearic acid methyl ester-d_{31}, (c): heptadecane-d_{31}. (d): standard palmitic acid-d_{31} and stearic acid (red) and extracts from cells without methylation (blue). (e): product from *in vitro* photocatalytic reaction using stearoyl-CoA as substrate and purified *Cv*FAP as the catalyst.

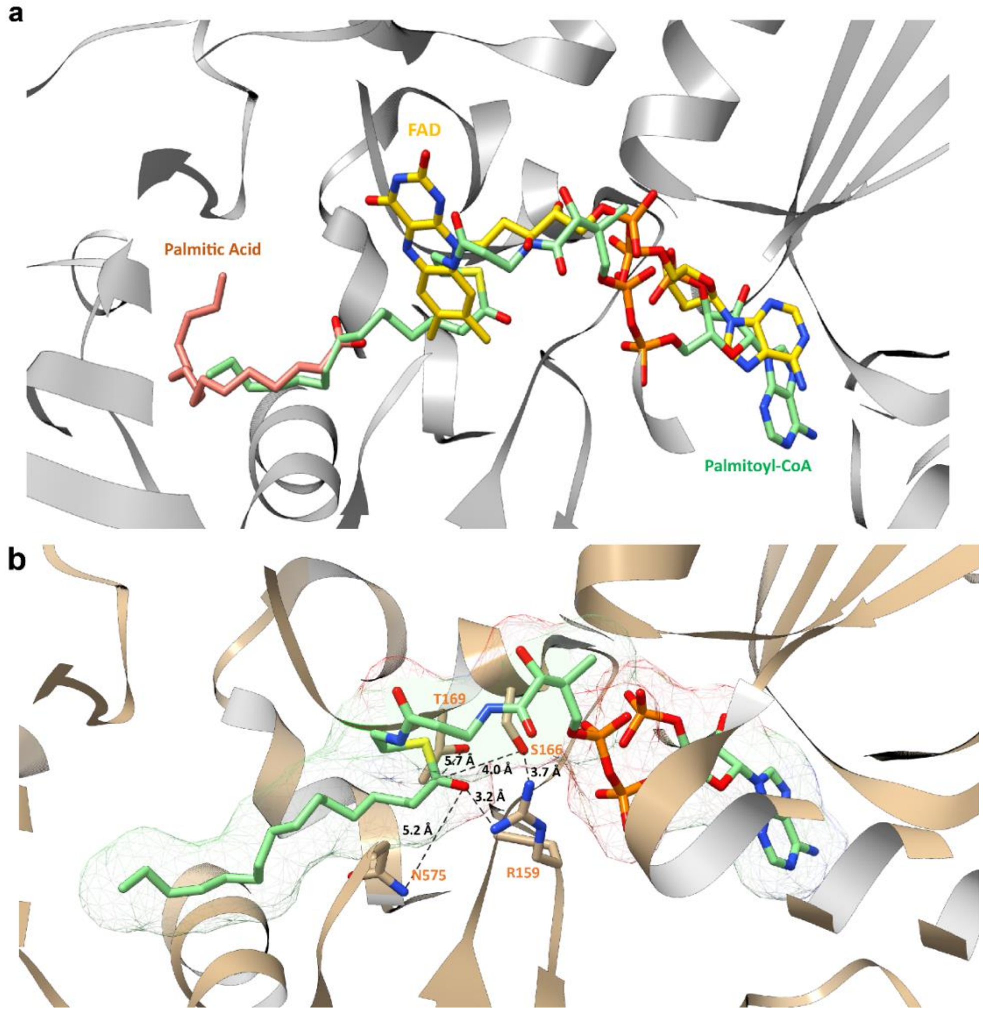

Supplementary Figure 4. Molecule docking of palmitoyl-CoA with *Cv*FAP. (a); Active site structure showing docked palmitoly-CoA molecule superimposed onto the original structure of *Cv*FAP (PDB ID: 5NCC) with palmitic acid and FAD bound. (b): Active site of *Cv*FAP docked with palmitoyl-CoA, showing the distances of polar residues in close proximity to acyl-CoA thioester carbonyl group. The distances from S166 and T169 are to the carbonyl carbon whereas the distances from R159 and N575 are to the carbonyl oxygen. According to this docking analysis, two mechanisms for hydrolysis might be suggested. Similar to CalE7 thioesterase [1], a water/hydroxide might carry out a nucleophilic attack on the carbonyl carbon and R159 (at 3.2 Å distance to the oxygen) can act as an oxyanion hole to stabilize the intermediate. Another possible mechanism is that, similar to many other thioesterases, S166 (at 4.0 Å distance to carbonyl carbon) can act as a nucleophile. The nearby R159 (at 3.7 Å distance) can possibly act as a base to activate S166, if the p*K*a of R159 is somewhat lowered by its local surroundings. The involvement of other polar residues, such as T169, cannot be ruled out considering the likelihood of conformational differences between an acyl-CoA bound *Cv*FAP and fatty acid/FAD bound *Cv*FAP structure, which will bring other polar residues closer to the thioester bond. Revealing the exact mechanism will require the structure of *Cv*FAP with acyl-CoA bound.

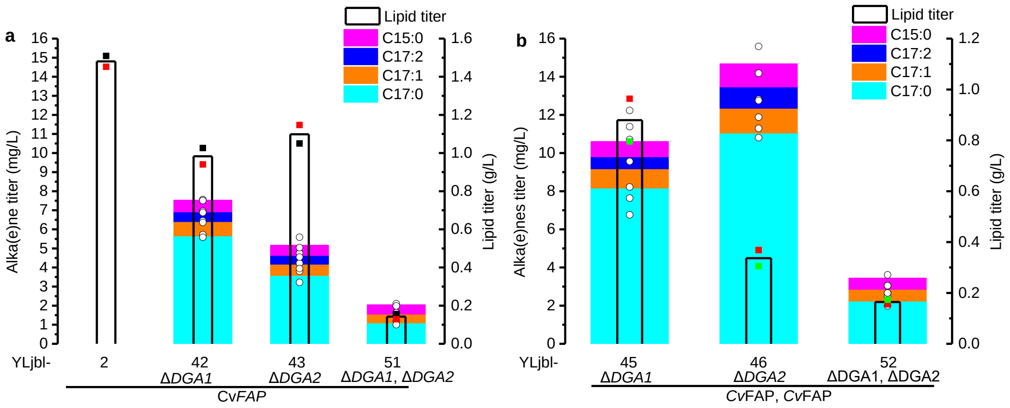

Supplementary Figure 5. The effect of DGA knockouts on lipid and alka(e)ne titer. (a) The effect of deletions of *DGA1* and *DGA2* and (b) The effect of increasing the Cv*FAP* copy number on regulating lipid and alka(e)ne synthesis. Fermentations were performed in 50 mL conical shake flasks with 3 days in dark followed by 3 days in blue light generated by light source 1. The working volume and initial OD_{600} was 13 mL and 0.1, respectively. Data represent mean value, n=2 biologically independent samples. Source data are provided as a Source Data file.

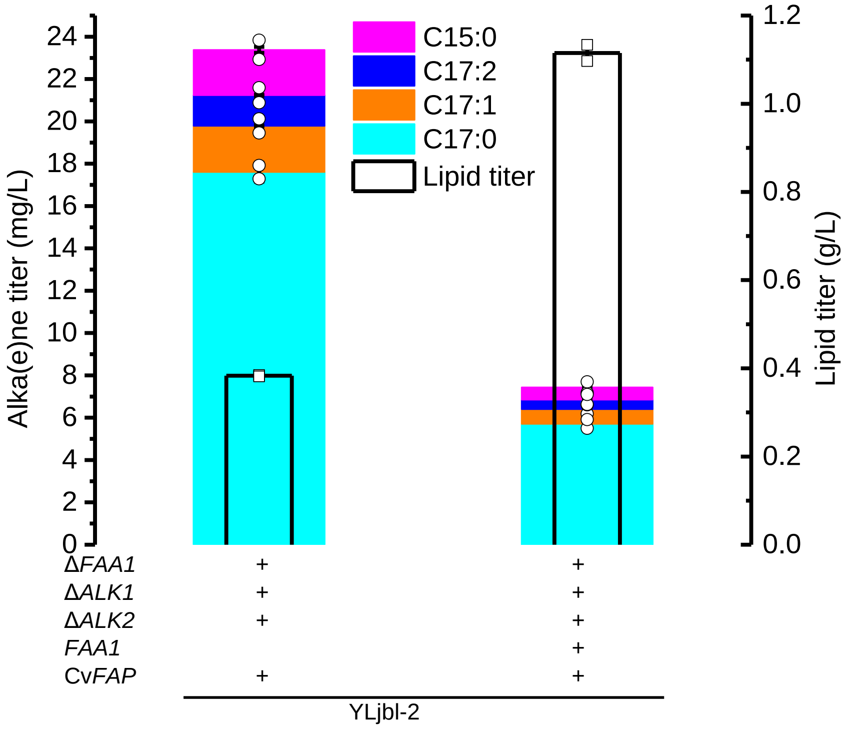

Supplementary Figure 6. The function of FAA1 in regulating lipid and alka(e)ne synthesis. Fermentations were performed in 50 mL conical shake flasks with 3 days in dark followed by 3 days in blue light generated by light source 1. The working volume and initial OD_{600} was 13 mL and 0.1, respectively. Data represent mean value ± SD, n=2 biologically independent samples. Source data are provided as a Source Data file.

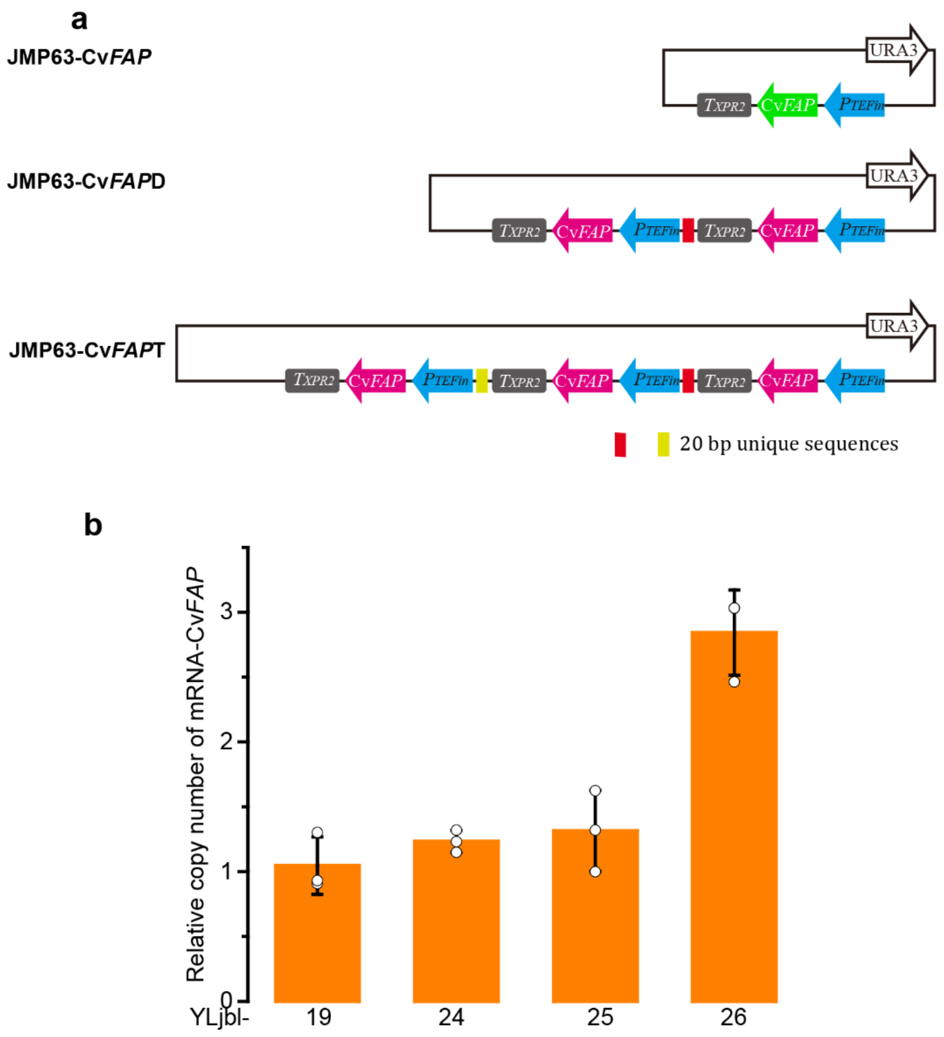

Supplementary Figure 7. Method to increase CvFAP copy number and its quantification. (a): Schematic of plasmids containing different Cv*FAP* copies. 20 bp unique sequences (red and yellow) were inserted for Gibson assembly. (b): Relative copy number of mRNA-Cv*FAP* determined by reverse transcription–qPCR (n=3 biologically independent samples). Data represent mean value ± SD. Source data underlying Supplementary Figure 7b are provided as a Source Data file.

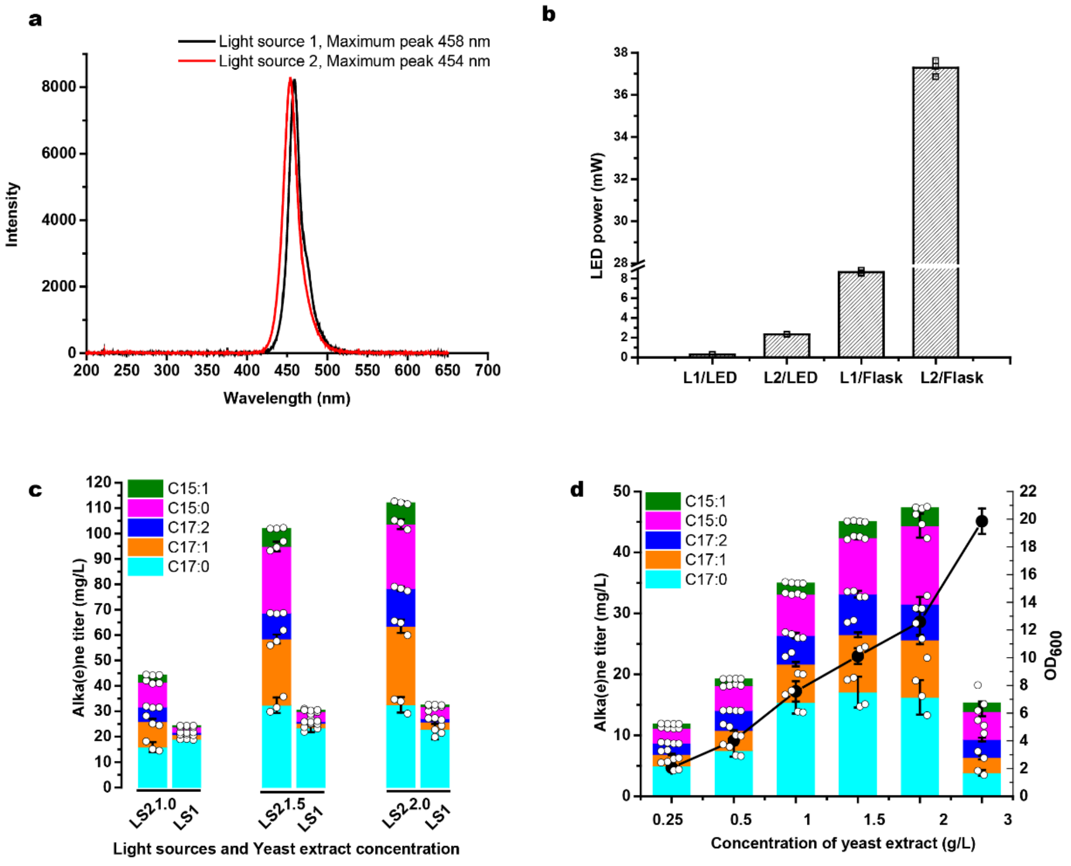

Supplementary Figure 8. Optimization of light source and nitrogen loading. (a): The spectra of both light sources. (b): The power of both light sources per bulb and per flask (n=3 biologically independent samples). (c): The effect of light sources and C/N ratios on alka(e)ne production (n=3 biologically independent samples). Fermentations were performed in 50 mL conical shake flasks with 2 days in dark followed by 1 day in blue light. The working volume and initial OD_{600} was 13 mL and 0.1, respectively. (d): The effect of nitrogen source on alka(e)ne production (n=3 or 4 biologically independent samples). Fermentations were performed in 12 well microplates with the working volume of 2 mL and an initial OD_{600} of 0.5. Samples were collected after 2 days cultivation in dark at 30 °C followed by 1-day cultivation in blue light generated by light source 2 at 25 °C. Data represent mean value ±SD. Source data underlying Supplementary Figure 8b-d are provided as a Source Data file.

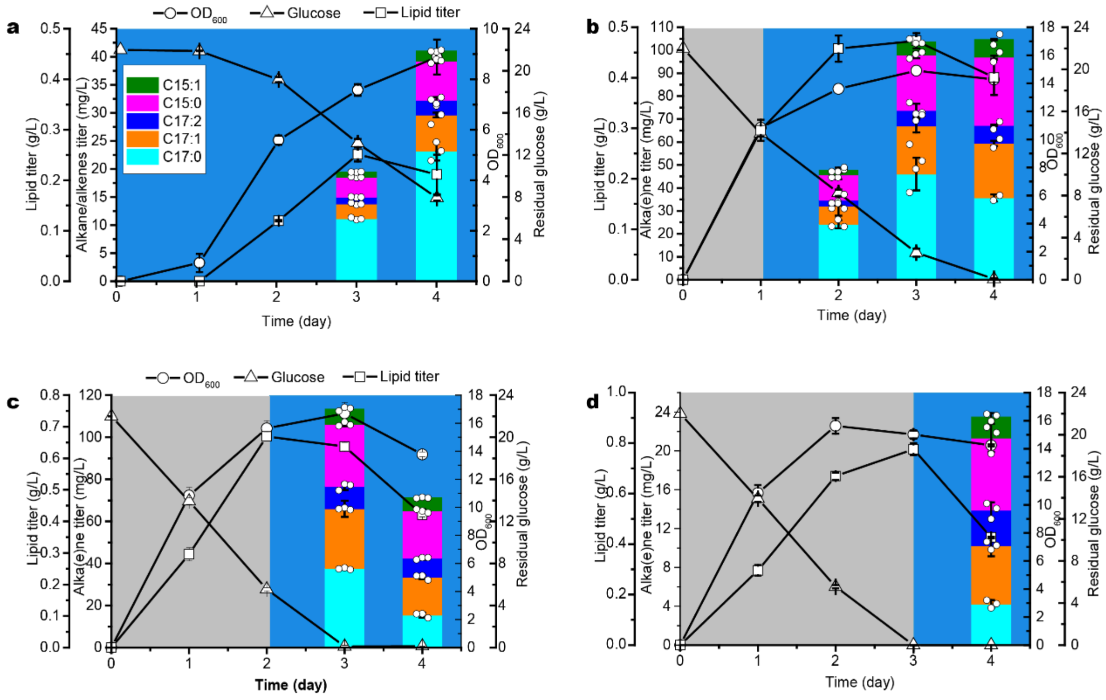

Supplementary Figure 9. The effects of switching point on alka(e)ne production, lipid production, and cell growth. Fermentations were performed in 50 mL conical shake flasks with working volume and initial OD_{600} of 13 mL and 0.1, respectively. Grey colored background indicates cultivation in dark at 30 °C while blue colored background indicates cultivation in blue light generated by light source 2 at 25 °C. Data represent mean value ±SD, n=3 biologically independent samples. Source data are provided as a Source Data file.

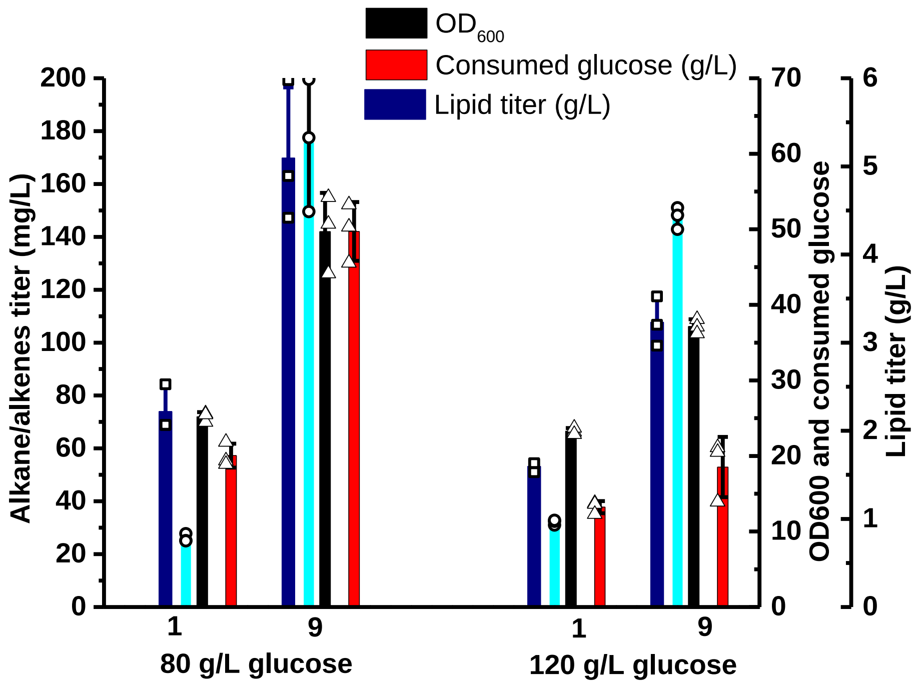

Supplementary Figure 10. Alka(e)ne titer resulting from batch fermentation with different initial glucose concentration. The C/N ratio was fixed at 43. Data points from 1-day or 9-day blue light illumination are shown. Fermentation was performed in 50 mL glass conical shake flasks with a working volume of 13 mL and initial OD_{600} of 0.1. The blue light was generated by light source 2. Data represent mean value ± SD, n=3 biologically independent samples. Source data are provided as a Source Data file.

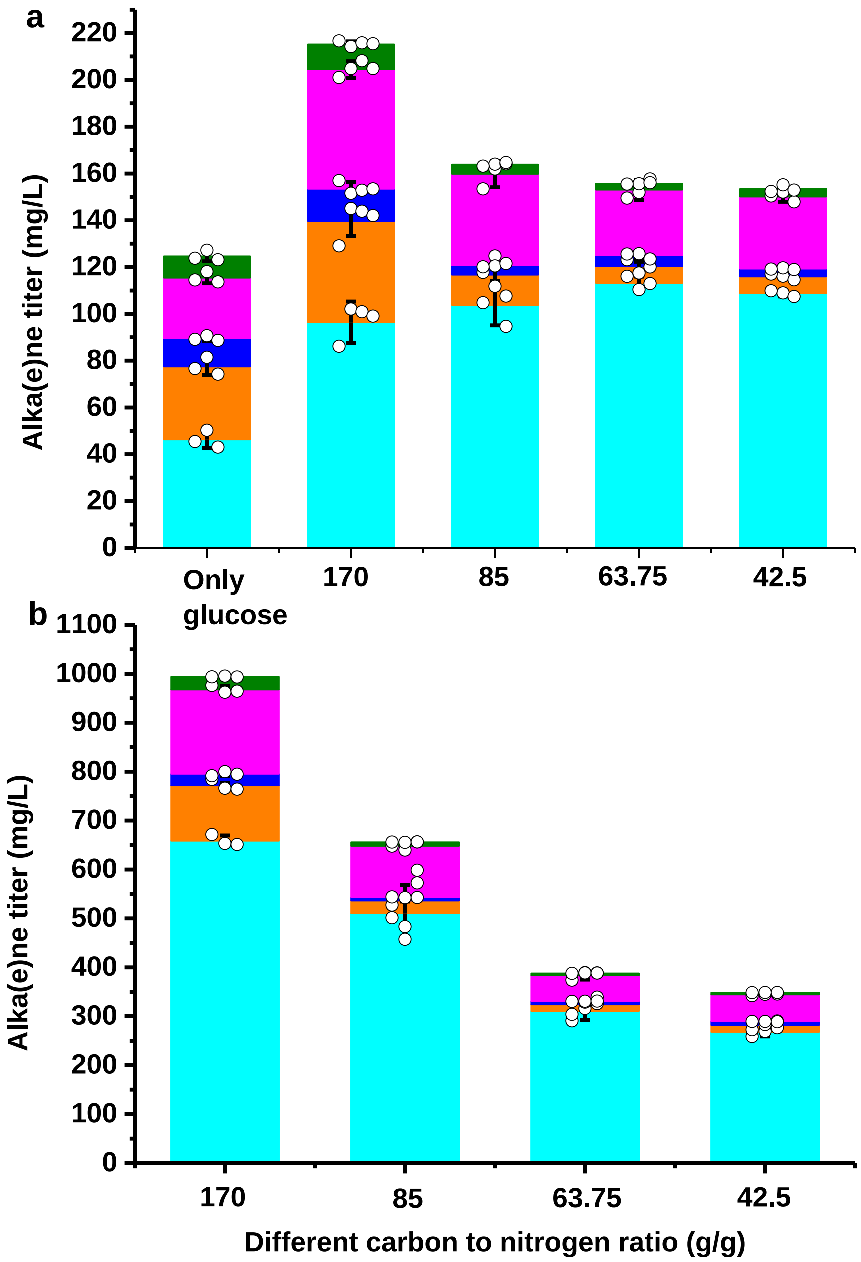

Supplementary Figure 11. Alka(e)ne titer resulting from fed-batch fermentation feeding with media of different C/N ratios. (a) Results after 2 days in dark and 2 days in light. (b) Results after 2 days in dark and 11 days in light. The fermentation strategy was exactly the same as shown in Fig. 4a. The blue light was generated by light source 2. Data represent mean value ±SD, n=3 or 4 biologically independent samples. Source data are provided as a Source Data file.

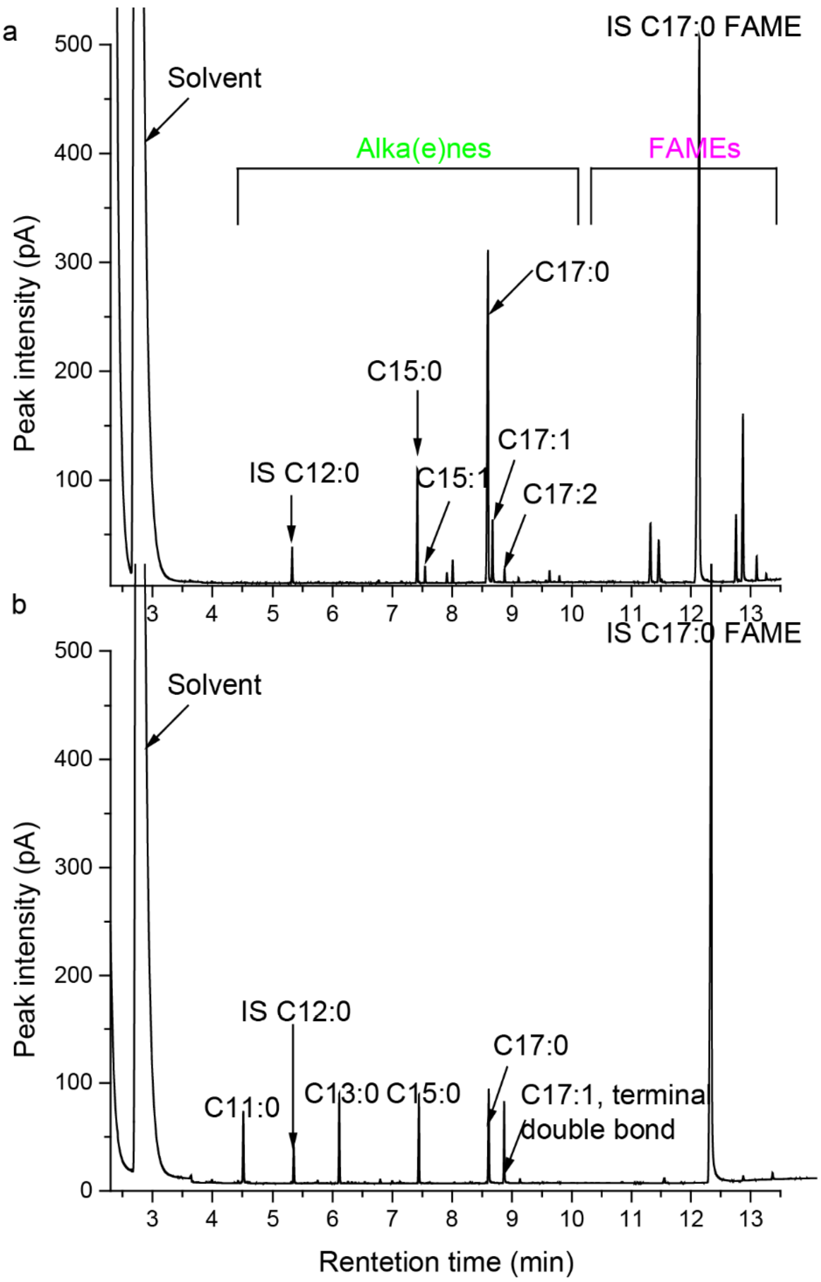

Supplementary Figure 12. Chromatograms of culture sample and standard compounds. (a) GC-FID chromatogram of alka(e)nes and FAMEs from cells shown in Fig. 4 with alka(e)ne titer of 1.03 g/L. IS represents internal standards which are dodecane and heptadecanoic acid methyl ester in this case. The chromatogram clearly shows the signal/peak areas of alka(e)nes overweigh FAMEs. (b) GC-FID chromatogram of standard alka(e)nes including C11:0, C12:0, C13:0, C15:0, C17:0, C17:1 (terminal double bond) and C17:0 FAME.

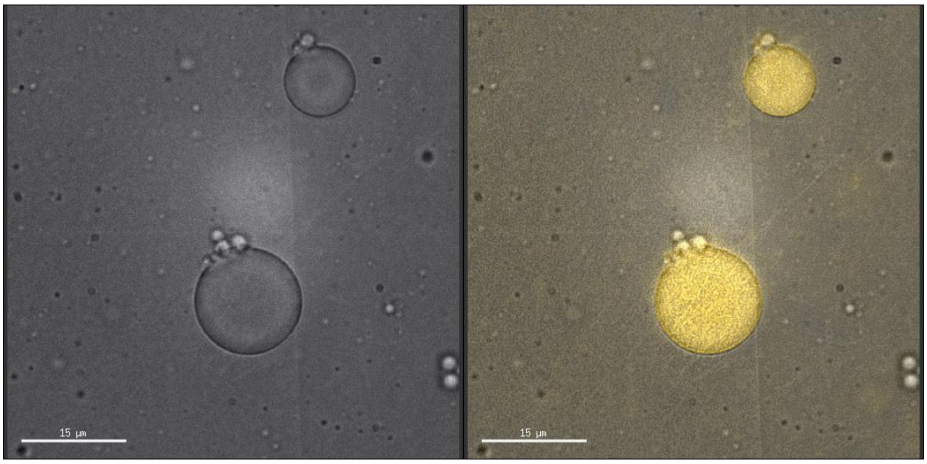

Supplementary Figure 13. Microscopic photos of the emulsion encapsulating the artificial mixture of heptadecane, heptadecene, and pentadecane stained by Nile red. The emulsion was prepared by mixing heptadecane, heptadecene, and pentadecane with water and Tween 80. The mixture was vigorously vortexed at 1000 rpm for 5 min before staining. The experiment was repeated at least 3 times and showed similar results.

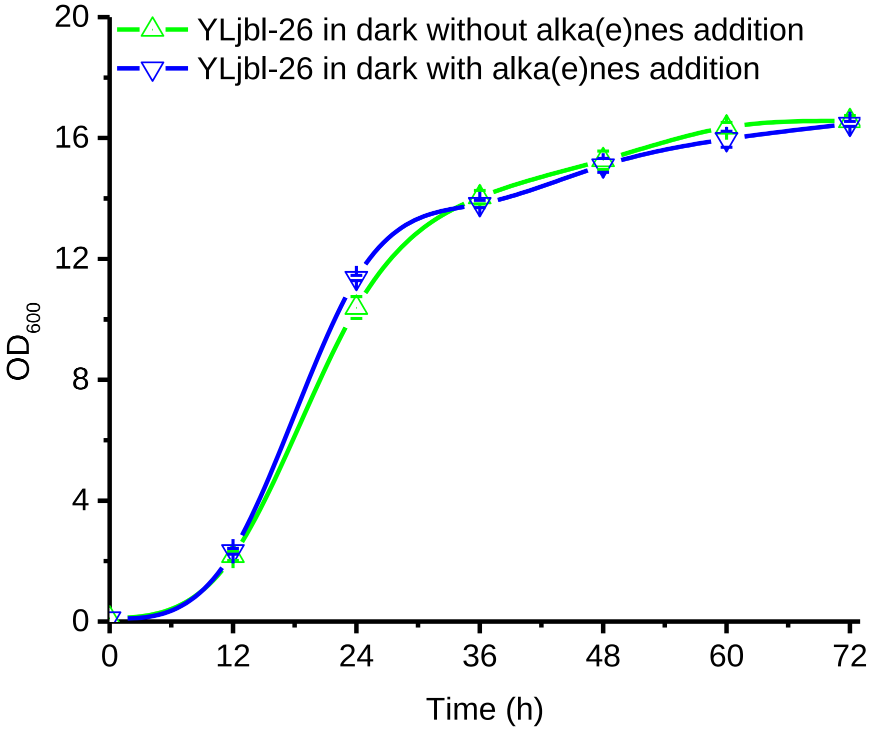

Supplementary Figure 14. Growth curve of producing strain in dark with/without the addition of alka(e)nes. The final concentration of 1.6 g/L alka(e)nes was added. Data represent mean value ±SD, n=3 biologically independent samples. Source data are provided as a Source Data file.

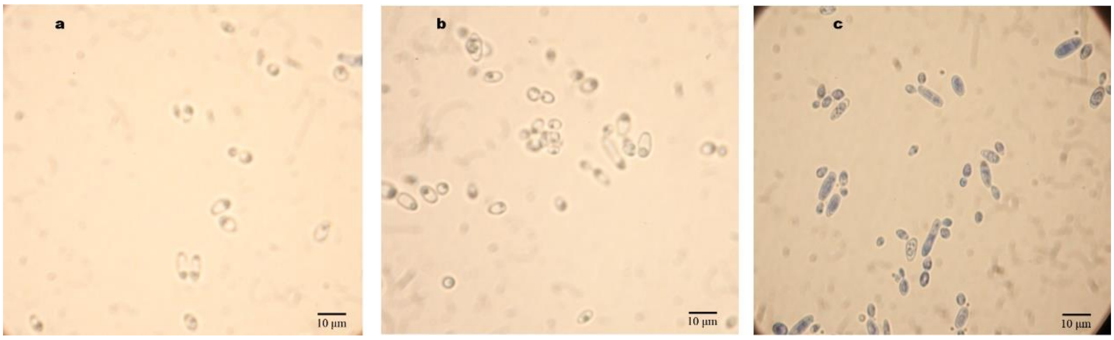

Supplementary Figure 15. Staining by trypan blue solution to check viable cells. (a): strain YLjbl-26 cultured in dark for 63 h. (b): strain YLjbl-26 cultivated in dark for 48 h, followed by cultivation in blue light for 15 h. The titer of alka(e)nes was 86.18 mg/L. (c): Negative control. Strain incubated at 50 °C for 1 h and stained by trypan blue. It is clear that cells in (c) were stained and showed blue color while the cells in (a) and (b) were not, indicating that cells in (a) and (b) were viable. The experiment was repeated at least 3 times and showed similar results.

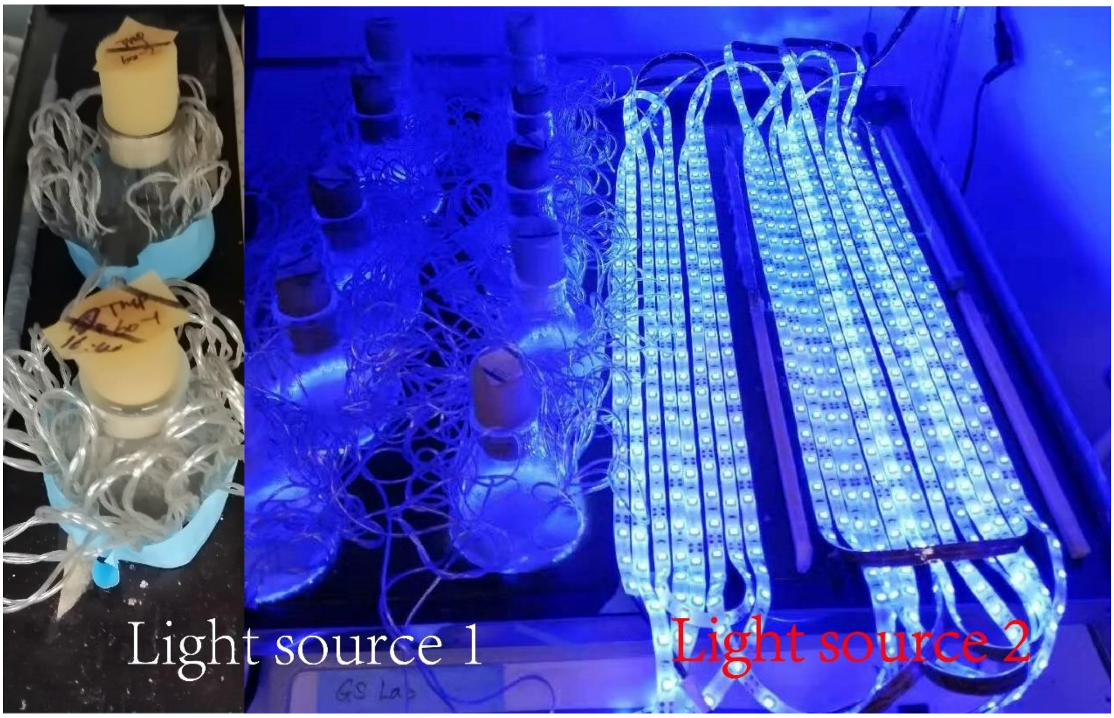

Supplementary Figure 16. The lab-setup of the light incubator with two different light sources. The characterization of both light sources could be found in Supplementary Figure 7. Photos were taken by J. Li.

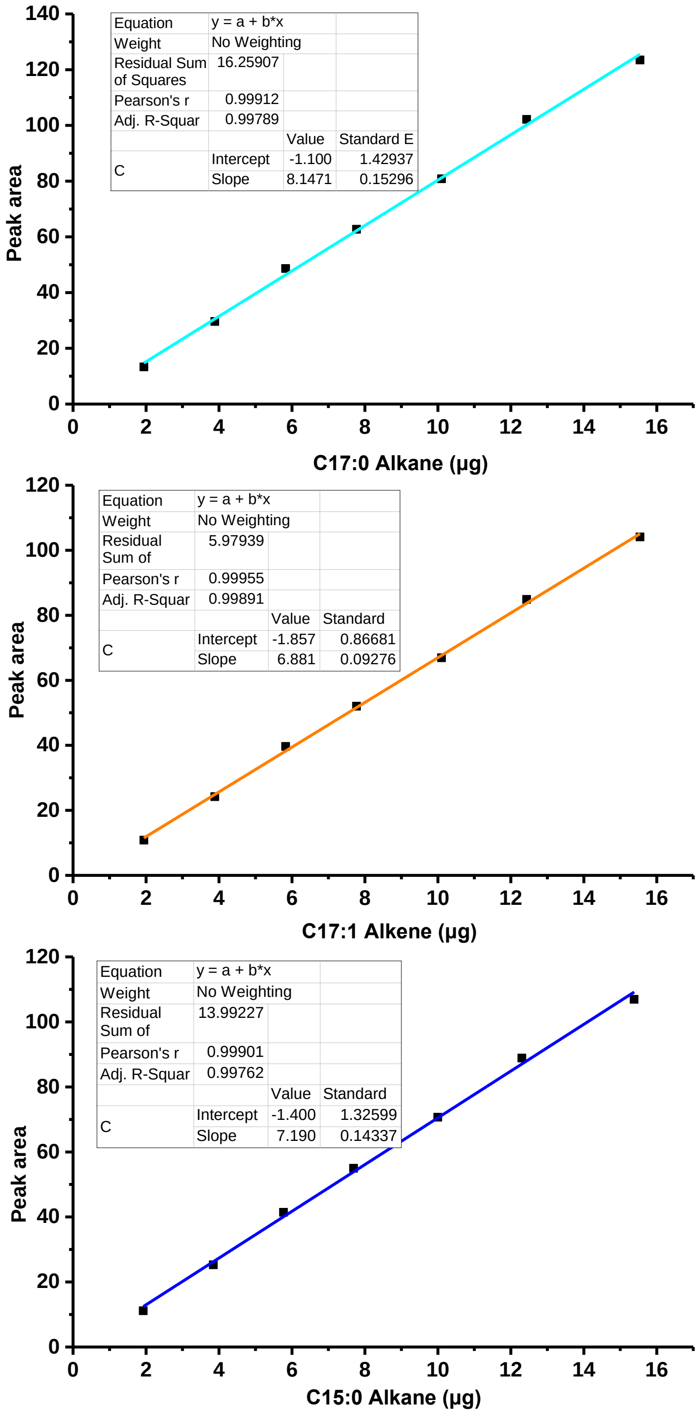

Supplementary Figure 17. Standard curves of alka(e)nes. Standard C17:1 alkene is a terminal alkene which is not the same as n-8 C17:1. Due to a lack of standard compounds, C15:1 and C17:2 were calculated as C15 and C17:1 equivalence,

respectively. All data points were normalized by the peak area of internal standard dodecane. Source data are provided as a Source Data file.

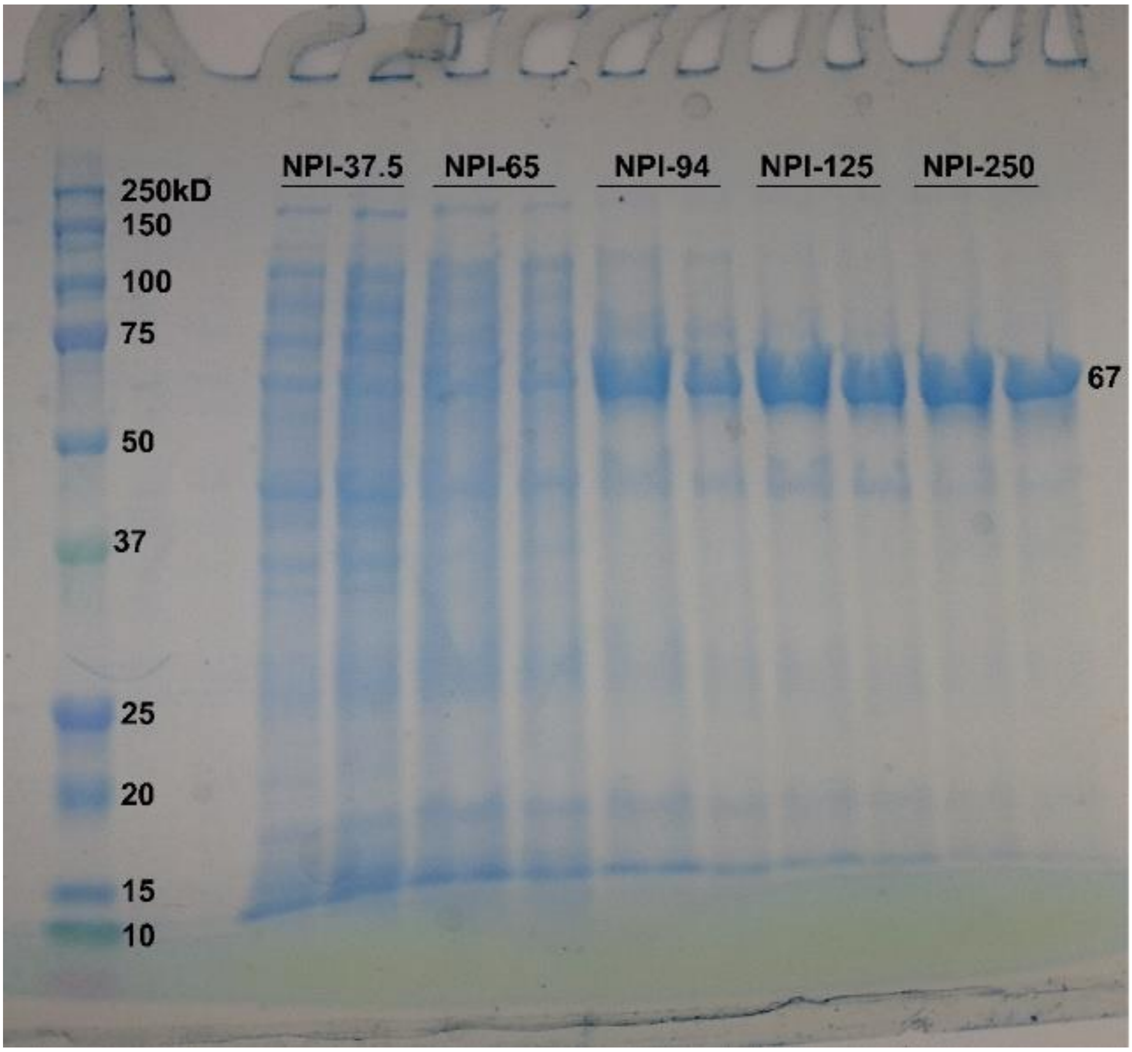

Supplementary Figure 18. SDS-PAGE of *Cv*FAP protein eluted by different concentrations of imidazole. The molecular weight of pure *Cv*FAP plus eight histidines is 67 kDa. Fractions NPI-125 and NPI-250 were combined and subjected to buffer exchange. The experiment was repeated at least 2 times and showed similar results.

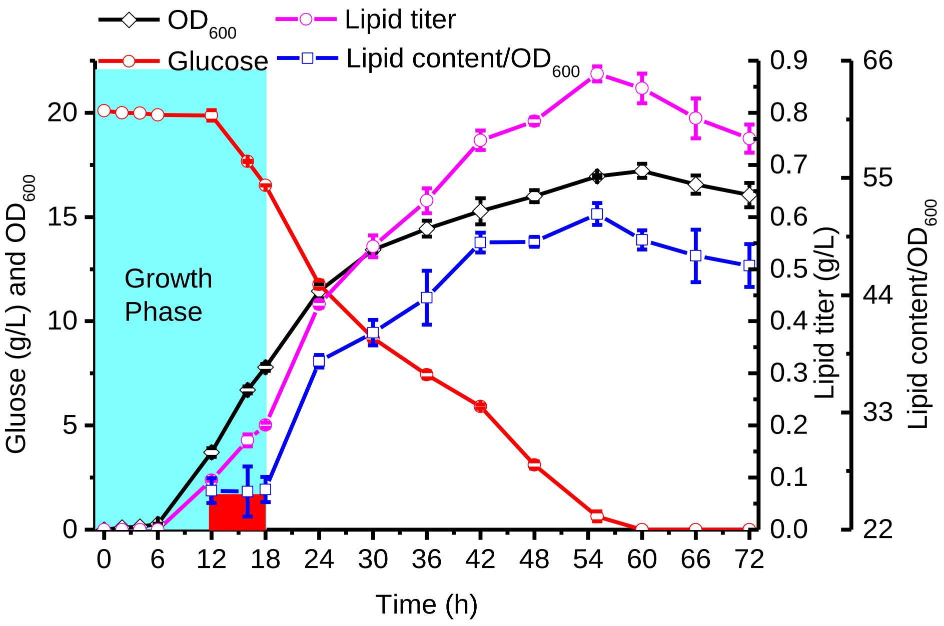

Supplementary Figure 19. Time course of YLjbl-26 in terms of glucose consumption, growth, lipid synthesis, and lipid content per OD_{600}. The cultivation was performed in YEM medium consisting of 20 g/L glucose, 6.9 g/L YNB-AA^{-}SA^{-}, and 2.0 g/L yeast extract. The first 18 h was defined as the growth phase due to the unchanged lipid content/OD_{600}. After 18 h, it entered into the lipid accumulation phase. Data represent mean value ±SD, n=3 biologically independent samples. Source data are provided as a Source Data file.

Supplementary Note 1. Proposed mechanism for the reaction of *Cv*FAP with fatty acyl-CoA to form alka(e)nes.

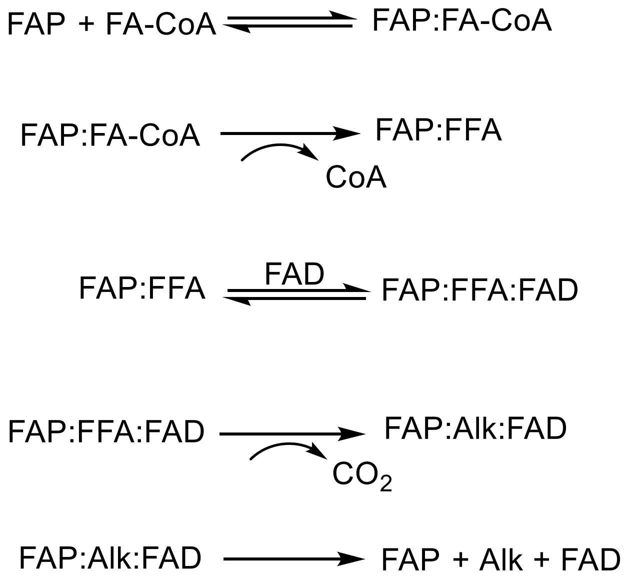

Supplementary Note 2: Calculation of percentage of acyl-CoA redirected to alka(e)nes

Fact 1: the method for quantifying lipid content in this work can transform fatty acyl moieties from both neutral lipids and polar lipids into fatty acid methyl esters [2]. Therefore, lipid content in the current work includes acyl chains from both neutral lipids and polar lipids.

Fact 2: at the early growth stage, *Y. lipolytica* does not accumulate lipids (Supplementary Figure 19, red indicated). The lipids detected during this period are considered as basic lipid requirements for viable cells.

Assumption 1: each viable cell has the same basic lipid requirement.

Assumption 2: each OD_{600} represents the same concentration of cells.

Based on the above, we calculated the minimum lipid content required per OD_{600} by adopting data points from 12, 16, and 18 h. The average minimum of lipid content/OD_{600} was 25.61 mg/L/OD_{600}.

With this, the following formula was generated.

$$\text{Percentage of acyl} - \text{CoA redirected to alka(e)nes (\%)} = \frac{\frac{Alka(e)ne\ titer}{AMW_{alk}}}{\frac{Lipid\ titer - 25.61 \times OD_{600}}{AMW_{fame}}} \times 100$$

where AMW_{alk} and AMW_{fame} are the average molecular weight of alka(e)nes and lipids of 233.38 and 285.14, respectively. The calculation of AMW is based on the composition of fatty acid methyl esters and alka(e)nes and their corresponding molecular weight.

Supplementary Table 1. Summary of microbial production of hydrocarbons

Chain length	Host	Pathway for alkane production	Energy source	Cultivation condition	Titer	Yield	Ref
C15-C17	*Y. lipolytica*	Photo-driven decarboxylation pathway	Photons from blue light	SMM+glucose (80 g/L), shake flask	1.03 g/L	12.34 mg/g[a]	This work
C15-C17	*Y. lipolytica*	Photo-driven decarboxylation pathway	Photons from blue light	SMM+glucose (120 g/L) hydrolysate, shake flask	1.20 g/L	9.64 mg/g[a]	This work
C15-C17	*Y. lipolytica*	Photo-driven decarboxylation pathway	Photons from blue light	SMM+glucose (160 g/L) hydrolysate, shake flask	1.47 g/L	8.88 mg/g[a]	This work
C15-C17	*Y. lipolytica*	Photo-driven decarboxylation pathway	Photons from blue light	SMM+acetate, shake flask	318.7 mg/L	5.72 mg/g[b]	This work
C15-C17	*Y. lipolytica*	Photo-driven decarboxylation pathway	Photons from blue light	SMM+wheat straw hydrolysate, shake flask	242.04 mg/L	4.66 mg/g[a]	This work
C15-C17	*Y. lipolytica*	Photo-driven decarboxylation pathway	Photons from blue light	SMM+glucose or glycerol, fed-batch	58.7 mg/L	NG	[3]
C13-C17	*Y. lipolytica*	Cyanobacterial pathway	NADPH	MM+glucose, shake flask	23 mg/L	0.39 mg/g	[4]
C13-C17	*E. coli*	Cyanobacterial pathway	NADPH	MM+glucose, shake flask	0.30 g/L	10 mg/g	[5]
C15, C17	*E. coli*	Cyanobacterial pathway	NADPH	MM+glycerol, shake flask	1.31 g/L	11 mg/g	[6]
C9-C13	*E. coli*	Cyanobacterial pathway	NADPH	MM+glucose, shake flask	0.58 g/L	NC	[7]
C3, C4, C5	*E. coli*	Cyanobacterial pathway	NADPH	LB+glycerol, shake flask	1.3-4.3 mg/L	NC	[8]
C3	*E. coli*	Cyanobacterial pathway	NADPH	MM+glucose, batch	32 mg/L	0.43 mg/g	[9]

C13-C17	*E. coli*	$OleT_{JE}$ P450 fatty acid decarboxylase powered pathway	NADPH	MM+glucose, shake flask	97 mg/L	3.2 mg/g	[10]
C13-C17	*S. cerevisiae*	Cyanobacterial pathway	NADPH	MM+glucose, shake flask	3.6 mg/L	0.12 mg/g	[11]
C11-C19	*S. cerevisiae*	Desaturase-like enzyme UndB powered decarboxylation pathway	Not clear yet	MM+glucose, shake flask	35.3 mg/L	1.8 mg/g	[12]

MM: minimal medium. SMM: semi-minimal medium. [a]: based on the consumed glucose plus yeast extract. [b]: based on the consumed acetic acid equivalence plus yeast extract. NG: not given.

Supplementary Table 2. Plasmids used and constructed in the study

Plasmids	Characteristics	Source
pJMP62 (LEU2)	KanR, LEU2 marker, TEF promoter and XPR2 terminator	Lab stock
pJMP63 (URA3)	KanR, URA3 marker, TEF-in promoter and XPR2 terminator	Lab stock
pPMT91 (URA3)	CmR, URA3 marker, TEF-in promoter and XPR2 terminator	Lab stock
pCRISPR (URA3)	AmpR, URA3 marker, UAS1B-TEF promoter and CYC1 terminator	[13]
pJMP63-Cv*FAP*	pJMP63 carrying Cv*FAP* from *Chlorella variabilis*	This study
pJMP63-*FAA1*	pJMP63 carrying *FAA1* from *Y. lipolytica*	This study
pPMT91-Ec*TesA'*	pPMT91 carrying *E. coli* truncated thioesterase TesA'	This study
pPMT91-Uc*ACPT*	pPMT91 carrying Uc*ACPT* from *Umbellularia californica*	This study
pPMT91-Ec*TesA'*-ER	pPMT91 carrying *E. coli* truncated thioesterase TesA' with ER retention signal KDEL	This study
pPMT91-Uc*ACPT*-ER	pPMT91 carrying Uc*ACPT* with ER retention signal KDEL	This study
pPMT91-*FAS*-Ec*TesA'*	pPMT91 carrying hybrid *Y. lipolytica FAS1* and Ec*TesA'*	This study
pPMT91-*FAS*-Uc*ACPT*	pPMT91 carrying hybrid *Y. lipolytica FAS1* and Uc*ACPT*	This study
pJMP63-*TGL3*-*TGL4*	pJMP63 carrying *TGL3* and *TGL4* from *Y. lipolytica*	This study
pJMP63-Cv*FAP*D	pJMP63 carrying duplicate Cv*FAP* expression cassette	This study
pJMP63-Cv*FAP*T	pJMP63 carrying triplicate Cv*FAP* expression cassette	This study

Supplementary Table 3. Strains used and constructed in this study

Strain	Genotype	Source
Po1f	*MatA, leu2-270, ura3-302, xpr2-322, axp-2*	[14]
YLjbl-1[a]	*MatA, leu2-270, LEU2, ura3-302, xpr2-322, axp-2*	This study
YLjbl-2	YLjbl-1-Cv*FAP*	This study
YLjbl-5	YLjbl-2-Ec*TesA'*	This study
YLjbl-6	YLjbl-2-Ec*TesA'-ΔFAA1*	This study
YLjbl-7	YLjbl-2-Uc*ACPT*	This study
YLjbl-8	YLjbl-2-Uc*ACPT-ΔFAA1*	This study
YLjbl-9	YLjbl-2-*TGL3-TGL4*	This study
YLjbl-10	YLjbl-2-Ec*TesA'-ER-ΔFAA1*	This study
YLjbl-11	YLjbl-2-Uc*ACPT-ER-ΔFAA1*	This study
YLjbl-12	YLjbl-2-*FAS*- Ec*TesA'-ΔFAA1*	This study
YLjbl-13	YLjbl-2-*FAS*-Uc*ACPT-ΔFAA1*	This study
YLjbl-14	YLjbl-2-Cv*FAP*	This study
YLjbl-15	YLjbl-2-Cv*FAP, ΔALK1*	This study
YLjbl-16	YLjbl-2-Cv*FAP, ΔALK2*	This study
YLjbl-17	YLjbl-2-Cv*FAP, ΔALK1 ΔALK2*	This study
YLjbl-18	YLjbl-2-Cv*FAP, ΔALK1 ΔFAA1*	This study
YLjbl-19	YLjbl-2-Cv*FAP, ΔALK2 ΔFAA1*	This study
YLjbl-20	YLjbl-2-Cv*FAP, ΔALK1 ΔALK2 ΔFAA1*	This study
YLjbl-21	YLjbl-20-Cv*FAP*	This study
YLjbl-22	YLjbl-20-Cv*FAP*D	This study
YLjbl-23	YLjbl-20-Cv*FAP*T	This study
YLjbl-24	YLjbl-19-Cv*FAP*	This study
YLjbl-25	YLjbl-19-Cv*FAP*D	This study
YLjbl-26	YLjbl-19-Cv*FAP*T	This study
YLjbl-42	YLjbl-2, *ΔDGA1*	This study
YLjbl-43	YLjbl-2, *ΔDGA2*	This study
YLjbl-51	YLjbl-2, *ΔDGA1, ΔDGA2*	This study
YLjbl-45	YLjbl-42-Cv*FAP*	This study
YLjbl-46	YLjbl-43-Cv*FAP*	This study

YLjbl-52	YLjbl-51-Cv*FAP*	This study

[a]: Leu+ derivative of po1f obtained by integration of empty pJMP62.

Supplementary Table 4. Primers, homologous arms, and gRNAs for knockouts. All were synthesized by Sigma.

	Primer name	Sequence (5’>3’)
FAA1	HR	GTATTCCCATTGTCACTGCCTACGACACTCTTGGAGAGGAGGGTCTCACTCACACATTTTCGAGTTTGTGTTCGAGATGGTCACCTTCTGGTGGGGTGCT
	gRNA	CCGAGAACACCCCCACCCCG
	Colony-f	CCACCGTCTACAAGCTGTTC
	Colony-r	CTTGAAAATGAGCGAGTCGA
ALK1	HR	TCTGTCGGTTCCCAGACCGTCGAAACCAACCCCACTGCCGTCCCCACCGAGTTCTTAACGAAGTCCTGCGACTCTACCCCTCTGTCCCTGCCAACATGCGA
	gRNA	TTTCCTCCGAGAGCTTGCCA
	Colony-f	GATGGCTCTGCCTTTGATGC
	Colony-r	ACGTGGTATGAAACGAGGTT
ALK2	HR	GTTCGACATCCAGGAGCTCTTCTTCAACCTGACCCTTGATACTGCCACCGTGTCCTGGATCGTCTACGAGCTGGCCCGACACCCCGAGGTGTGGAAGAAG
	gRNA	CTGAGCTCTTCTCTTCCTCC
	Colony-f	GCACTCTCGAGCCATGCT
	Colony-r	GATGACAAATCGCAGGTACT

DGA1	HR	TGGATCTGGAGCCCACGCACACATACTACCCTCTGGACGTCCAGGAGTATCCAAGCTCTTTCCGGGCATCCCTGTTTCTCTTATGACTCTCACCAACAAC
	gRNA	TTCAGATGACGCAGAGACTT
	Colony-f	TCCCACTTTCCTCACAATTT
	Colony-r	GACCTCCATACCAAGTCGAA
DGA2	HR	TACATGGCCGTGGGGCTTCTGCATACCATGAACACTTTGTCGTCCATCTCATCTTCCAGTACGCCTACCCCATCATGCAGTCGTGTCTGGCTCTGTTCTT
	gRNA	TTCAGATGACGCAGAGACTT
	Colony-f	CGGCATGATCATTCTCATTG
	Colony-r	GCTTGTTCCATAGAGTCCAG

Supplementary Table 5. Primers used in this study. All were synthesized by Sigma.

Primer name	Sequence (5’>3’)
QPCR-Cv*FAP*-F	CACACCCTTTCTGCTGAAGCAC
QPCR-Cv*FAP*-R	GAGATGGCGATTCCGTCGTACTT
ACT-RT-F	GAGTCACCGGTATCGTTC
ACT-RT-R	GCGGAGTTGGTGAAAGAG
Cv*FAP*D-F	CTGTTATCCCTAGAATCGATAGAGACCGGGTTGGCG
Cv*FAP*D-R	ACCAACGTTGGCGCGCCATgGGACACGGGCATCTCACT
Cv*FAP*T-F	GAGATGCCCGTGTCCcATGGatcgtagctagcgtacactgAGAGACCGGGTTGGCG
Cv*FAP*T-R	CACATGGGTACCAACGTTGGGGACACGGGCATCTCACT
TGL4-F	attcaaaggcgcgccacaATGTTCACCTCCAGAGTTTCCGA
TGL4-R	ttggggtcggctcgagaTTAGCACGAGTCAGAACAGTTCTC
TGL3-F	TTTTTGCAGTACTAACCGCAGAAAAGCCGCGTGGCCG
TGL3-R	CCGGCAACGTGGGGCCTAGGCTAGTTTTGTCGCTTGGTCTGGTT
Ec*TesA’*-F	TTTTGCAGTACtaaccgcagGCCGCTGACACCCTGCTGATC
Ec*TesA’*-R	GCAAGACCGGCAACGTGGGGttaAGAGTCGTGGTTCACCA
Uc*ACPT*-F	TATAAGAATCATTCAAAATGGTGAGTTTCAGAGGCAGCAG
Uc*ACPT*-R	GCAAGACCGGCAACGTGGGGTTAGACTCGGGGCTCGGCGG
Ylh*FAS*-F	TTTTGCAGTACtaaccgcaggtgagtatcgaccgaagcag
Ylh*FAS*-R	tccaggaagggatgcatagg

Supplementary References

1. Kotaka, M. *et al.* Structure and catalytic mechanism of the thioesterase CalE7 in enediyne biosynthesis. *J. Biol. Chem.* **284**, 15739–15749 (2009).
2. Liu, K. S. Preparation of fatty acid methyl esters for gas-chromatographic analysis of lipids in biological materials. *J. Am. Oil Chem. Soc.* **71**, 1179–1187 (1994).
3. Bruder, S., Moldenhauer, E. J., Lemke, R. D., Ledesma-Amaro, R. & Kabisch, J. Drop-in biofuel production using fatty acid photodecarboxylase from Chlorella variabilis in the oleaginous yeast *Yarrowia lipolytica*. *Biotechnol. Biofuels* **12**, 1–13 (2019).
4. Xu, P., Qiao, K., Ahn, W. S. & Stephanopoulos, G. Engineering *Yarrowia lipolytica* as a platform for synthesis of drop-in transportation fuels and oleochemicals. *Proc. Natl. Acad. Sci. U. S. A.* **113**, 10848–53 (2016).
5. Schirmer, A., Rude, M. A., Li, X., Popova, E. & del Cardayre, S. B. Microbial Biosynthesis of Alkanes. *Science* **329**, 559–562 (2010).
6. Cao, Y. X. *et al.* Heterologous biosynthesis and manipulation of alkanes in *Escherichia coli*. *Metab. Eng.* **38**, 19–28 (2016).
7. Choi, Y. J. & Lee, S. Y. Microbial production of short-chain alkanes. *Nature* **502**, 571–574 (2013).
8. Sheppard, M. J., Kunjapur, A. M. & Prather, K. L. J. Modular and selective biosynthesis of gasoline-range alkanes. *Metab. Eng.* **33**, 28–40 (2016).
9. Kallio, P., Pásztor, A., Thiel, K., Akhtar, M. K. & Jones, P. R. An engineered pathway for the biosynthesis of renewable propane. *Nat. Commun.* **5**, 4–11 (2014).
10. Liu, Y. *et al.* Hydrogen peroxide-independent production of α-alkenes by OleTJE P450 fatty acid decarboxylase. *Biotechnol. Biofuels* **7**, 28 (2014).
11. Zhou, Y. J. *et al.* Harnessing Yeast Peroxisomes for Biosynthesis of Fatty-Acid-Derived Biofuels and Chemicals with Relieved Side-Pathway Competition. *J. Am. Chem. Soc.* **138**, 15368–15377 (2016).
12. Zhou, Y. J., Hu, Y., Zhu, Z., Siewers, V. & Nielsen, J. Engineering 1-Alkene Biosynthesis and Secretion by Dynamic Regulation in Yeast. *ACS Synth. Biol.* **7**, 584–590 (2018).
13. Schwartz, C. M., Hussain, M. S., Blenner, M. & Wheeldon, I. Synthetic RNA Polymerase III Promoters Facilitate High-Efficiency CRISPR-Cas9-Mediated Genome Editing in *Yarrowia lipolytica*. *ACS Synth. Biol.* **5**, 356–359 (2016).
14. Madzak, C., Tréton, B. & Blanchin-Roland, S. Strong hybrid promoters and integrative expression/secretion vectors for quasi-constitutive expression of heterologous proteins in the yeast *Yarrowia lipolytica*. *J. Mol. Microbiol. Biotechnol.* **2**, 207–216 (2000).
