# Supplementary material for: Synthesis of high-titer alka(e)nes in Yarrowia lipolytica is enabled by a discovered mechanism
Source: Nat Commun. 2020 Dec 3;11:6198. doi: 10.1038/s41467-020-19995-0 (PMC7713262; doi:10.1038/s41467-020-19995-0)
Supplement: Supplementary file 2 — Description of Additional Supplementary Files [file 41467_2020_19995_MOESM2_ESM.pdf]

Description of Additional Supplementary Files

Supplementary Data 1:

Synthetic gene fragments used in this study. All synthetic genes were synthesized by Life technology gene art strings DNA.
