# Supplementary material for: Synthesis of high-titer alka(e)nes in Yarrowia lipolytica is enabled by a discovered mechanism
Source: Nat Commun. 2020 Dec 3;11:6198. doi: 10.1038/s41467-020-19995-0 (PMC7713262; doi:10.1038/s41467-020-19995-0)
Supplement: Supplementary file 3 — Supplementary Data 1 [file 41467_2020_19995_MOESM3_ESM.docx]

**Supplementary Data 1. Synthetic gene fragments used in this study**. All synthetic genes were synthesized by Life technology gene art strings DNA.

| Name | Sequence (5’>3’) |
| --- | --- |
| Cv*FAP* | ATGTCTGCCTCTGCCGTCGAGGACATCCGAAAGGTGCTGTCTGACTCTTCTTCGCCCGTGGCCGGACAGAAGTACGACTACATCCTGGTCGGCGGAGGCACCGCCGCCTGCGTGCTGGCCAACCGACTGTCTGCCGACGGCTCTAAGCGAGTGCTGGTGCTCGAGGCTGGCCCCGACAACACCTCTCGAGATGTGAAGATTCCCGCCGCTATTACCCGACTGTTCCGATCTCCTCTGGACTGGAACCTGTTCTCTGAGCTGCAAGAGCAGCTGGCCGAGCGACAGATCTACATGGCCCGAGGCCGACTGCTCGGCGGATCTTCTGCCACCAACGCTACCCTGTACCACCGAGGCGCCGCTGGCGACTACGACGCCTGGGGCGTCGAAGGCTGGTCCTCTGAGGACGTGCTGTCCTGGTTCGTGCAGGCCGAGACTAACGCCGACTTCGGACCCGGCGCTTACCACGGCTCTGGCGGACCCATGCGAGTCGAGAACCCTCGATACACCAACAAGCAGCTGCACACCGCTTTCTTCAAGGCCGCCGAGGAAGTGGGACTGACCCCTAACTCTGACTTCAACGACTGGTCCCACGACCACGCCGGCTACGGCACCTTCCAGGTGATGCAGGACAAGGGCACCCGAGCCGACATGTACCGACAGTACCTGAAGCCTGTGCTGGGCCGACGAAACCTCCAGGTGCTGACCGGCGCTGCCGTGACCAAGGTGAACATCGACCAGGCCGCTGGCAAGGCCCAGGCTCTGGGCGTCGAGTTCTCTACCGACGGACCCACCGGCGAGCGACTGTCCGCTGAGCTGGCTCCCGGCGGAGAGGTGATCATGTGCGCTGGCGCCGTGCACACACCCTTTCTGCTGAAGCACTCTGGCGTGGGCCCCTCTGCCGAGCTGAAGGAATTCGGCATCCCCGTGGTGTCTAACCTGGCCGGCGTCGGACAGAACCTGCAGGATCAGCCCGCCTGCCTGACCGCTGCTCCCGTGAAGGAAAAGTACGACGGAATCGCCATCTCTGACCACATCTACAACGAGAAGGGCCAGATCCGAAAGCGAGCCATTGCCTCTTACCTGCTCGGAGGCCGAGGCGGCCTGACCTCTACCGGCTGCGACCGAGGTGCCTTCGTGCGAACCGCCGGACAGGCTCTGCCCGATCTGCAGGTCCGATTCGTGCCCGGCATGGCTCTGGACCCCGACGGCGTGTCTACCTACGTGCGATTCGCCAAGTTCCAGTCTCAGGGCCTGAAGTGGCCCTCTGGCATCACCATGCAGCTGATCGCCTGTCGACCCCAGTCTACCGGATCTGTCGGACTGAAGTCTGCTGACCCCTTCGCTCCTCCTAAGCTGTCTCCCGGATACCTGACCGACAAGGACGGCGCCGACCTGGCTACCCTGCGAAAGGGCATCCACTGGGCCCGAGATGTGGCCCGATCTTCCGCTCTGTCTGAGTACCTGGACGGCGAGCTGTTCCCCGGCTCCGGCGTCGTGTCTGACGACCAGATCGACGAGTACATCCGACGATCTATCCACTCTTCTAACGCCATCACCGGCACCTGTAAGATGGGCAACGCCGGCGACTCTTCCTCTGTGGTGGACAACCAGCTGCGAGTCCACGGCGTCGAGGGCCTGCGAGTGGTGGACGCCTCCGTGGTGCCCAAGATTCCTGGCGGCCAGACTGGCGCTCCCGTGGTGATGATCGCCGAGCGAGCCGCCGCTCTGCTCACCGGCAAGGCCACCATCGGCGCCTCTGCTGCCGCTCCTGCCACCGTGGCCGCCTAA |
| Ec*TesA’* | ATGGCCGCTGACACCCTGCTGATCCTGGGCGACTCTCTGTCTGCCGGCTACCGAATGTCTGCCTCTGCCGCTTGGCCCGCTCTGCTGAACGACAAGTGGCAGTCTAAGACCTCTGTGGTGAACGCCTCTATCTCTGGCGACACCTCTCAGCAGGGCCTCGCTCGACTGCCTGCTCTGCTCAAGCAGCACCAGCCTCGATGGGTGCTCGTCGAGCTTGGCGGCAACGACGGCCTGCGAGGCTTCCAGCCTCAGCAGACCGAGCAGACCCTGCGACAGATTCTGCAGGACGTGAAGGCCGCCAACGCTGAGCCTCTGCTGATGCAGATCCGACTGCCCGCCAACTACGGCCGACGATACAACGAGGCCTTCTCTGCTATCTACCCCAAGCTGGCCAAGGAATTCGACGTGCCCCTGCTGCCATTCTTCATGGAAGAGGTGTACCTGAAGCCTCAGTGGATGCAGGACGACGGCATTCACCCCAACCGAGATGCTCAGCCCTTCATTGCCGACTGGATGGCCAAGCAGCTGCAGCCCCTGGTGAACCACGACTCTTAA |
| Uc*ACPT* | ATGGCCACCACCTCCCTGGCCTCCGCCTTCTGCTCCATGAAGGCCGTCATGCTGGCCCGAGACGGCCGAGGCATGAAGCCCCGATCCTCCGACCTGCAGCTGCGAGCCGGCAACGCCCCCACCTCTCTGAAGATGATCAACGGCACCAAGTTCTCCTACACCGAGTCCCTGAAGCGACTGCCCGACTGGTCCATGCTGTTCGCTGTCATCACCACCATCTTCTCCGCCGCCGAGAAGCAGTGGACCAACCTGGAGTGGAAGCCCAAGCCCAAGCTGCCCCAGCTGCTGGACGACCACTTCGGCCTGCACGGCCTGGTCTTCCGACGAACCTTCGCCATCCGATCTTACGAAGTCGGTCCCGACCGATCTACCTCCATTCTGGCCGTCATGAACCATATGCAGGAGGCTACCCTGAACCACGCCAAGTCCGTCGGCATCCTGGGCGACGGCTTCGGCACCACCCTGGAGATGTCCAAGCGAGACCTGATGTGGGTCGTCCGACGAACCCACGTCGCCGTCGAGCGATACCCCACCTGGGGCGACACCGTCGAGGTCGAGTGCTGGATCGGCGCCTCCGGCAACAACGGCATGCGACGAGACTTCCTGGTCCGAGACTGCAAGACCGGCGAGATCCTGACCCGATGCACTTCTCTCTCCGTCCTGATGAACACCCGAACCCGACGACTGTCCACCATCCCCGACGAGGTCCGAGGCGAGATCGGCCCCGCCTTCATCGACAACGTCGCCGTCAAGGACGACGAGATCAAGAAGCTGCAGAAGCTGAACGACTCCACCGCCGACTACATCCAGGGCGGCCTGACCCCCCGATGGAACGACCTGGACGTCAACCAGCACGTCAACAACCTGAAGTACGTCGCCTGGGTCTTCGAGACCGTCCCCGACTCCATCTTCGAGTCCCACCACATCTCCTCCTTCACCCTGGAGTACCGACGAGAGTGCACCCGAGACTCCGTCCTGCGATCCCTGACCACCGTCTCCGGCGGCTCCTCCGAGGCCGGCCTGGTCTGCGACCACCTGCTGCAGCTGGAGGGCGGCTCCGAGGTCCTGCGAGCCCGAACCGAGTGGCGACCCAAGCTGACCGACTCCTTCCGAGGCATCTCCGTCATCCCCGCCGAGCCCCGAGTCTAA |
