# Supplementary material for: Synthesis of high-titer alka(e)nes in Yarrowia lipolytica is enabled by a discovered mechanism
Source: Nat Commun. 2020 Dec 3;11:6198. doi: 10.1038/s41467-020-19995-0 (PMC7713262; doi:10.1038/s41467-020-19995-0)
Supplement: Supplementary file 4 — Reporting Summary [file 41467_2020_19995_MOESM4_ESM.pdf]

nature research

Corresponding author(s): Gregory Stephanopoulos

Last updated by author(s): Sep 19, 2020

Reporting Summary

Nature Research wishes to improve the reproducibility of the work that we publish. This form provides structure for consistency and transparency in reporting. For further information on Nature Research policies, see Authors & Referees and the Editorial Policy Checklist .

Statistics

For all statistical analyses, confirm that the following items are present in the figure legend, table legend, main text, or Methods section.

n/a	Confirmed	
☐	☒	The exact sample size (*n*) for each experimental group/condition, given as a discrete number and unit of measurement
☐	☒	A statement on whether measurements were taken from distinct samples or whether the same sample was measured repeatedly
☒	☐	The statistical test(s) used AND whether they are one- or two-sided<br>*Only common tests should be described solely by name; describe more complex techniques in the Methods section.*
☒	☐	A description of all covariates tested
☒	☐	A description of any assumptions or corrections, such as tests of normality and adjustment for multiple comparisons
☐	☒	A full description of the statistical parameters including central tendency (e.g. means) or other basic estimates (e.g. regression coefficient) AND variation (e.g. standard deviation) or associated estimates of uncertainty (e.g. confidence intervals)
☒	☐	For null hypothesis testing, the test statistic (e.g. *F*, *t*, *r*) with confidence intervals, effect sizes, degrees of freedom and *P* value noted<br>*Give P values as exact values whenever suitable.*
☒	☐	For Bayesian analysis, information on the choice of priors and Markov chain Monte Carlo settings
☒	☐	For hierarchical and complex designs, identification of the appropriate level for tests and full reporting of outcomes
☒	☐	Estimates of effect sizes (e.g. Cohen's *d*, Pearson's *r*), indicating how they were calculated

Our web collection on statistics for biologists contains articles on many of the points above.

Software and code

Policy information about availability of computer code

Data collection	Agilent OpenLab Software 7890B for HPLC, GC-FID, and GC-MS was used for collecting GC-FID, GC-MS, and HPLC data. ZEISS Axioskop EL-Einsatz with Nikon color camera was used to collect bright field microscopic data; DeltaVision2-TIRF Microscope was used to collect florescence images.
Data analysis	MS Excel 2016 MSO was used to process and analyze all data; Origin9 was used to plot all data; Chimera 1.13.1rc with ViewDock tool and DockPrep tool implemented and AutoDock Vina 1.1.2 were used for docking analysis.

For manuscripts utilizing custom algorithms or software that are central to the research but not yet described in published literature, software must be made available to editors/reviewers. We strongly encourage code deposition in a community repository (e.g. GitHub). See the Nature Research guidelines for submitting code & software for further information.

Data

Policy information about availability of data

All manuscripts must include a data availability statement. This statement should provide the following information, where applicable:
- Accession codes, unique identifiers, or web links for publicly available datasets
- A list of figures that have associated raw data
- A description of any restrictions on data availability

Source data that support figures 1b, 2e, 2f, 2g, 3b, 3c, 3d, 3f, 4a, 4b, 4c, 4d, ,5b, 5c, 5d, S2, S5, S6, S7b, S8b, S8c, S8d, S9a, S9b, S9c, S9d, S10, S11a, S11b, S14, S17, and S19 are provided. The data for plotting chromatograms and MS spectra are available upon request from corresponding author. Structure of fatty acid photodecarboxylase in complex with FAD and palmitic acid is available from Protein Data Base with ID: 5NCC (10.2210/pdb5NCC/pdb). Gene sequences are provided in the Supplementary Data file.

Field-specific reporting

Please select the one below that is the best fit for your research. If you are not sure, read the appropriate sections before making your selection.

☒ Life sciences ☐ Behavioural & social sciences ☐ Ecological, evolutionary & environmental sciences

For a reference copy of the document with all sections, see nature.com/documents/nr-reporting-summary-flat.pdf

Life sciences study design

All studies must disclose on these points even when the disclosure is negative.

Sample size	As engineering was performed at the genetic level, and metabolite titers represent a large population of individual cells, sample size (n=2-4) was determined based on the consistency of measurable differences between groups, where biological replicates represent independently grown microbial cultures.
Data exclusions	No data exclusion in this study.
Replication	2-4 biologically independent replicates were used to conform the reproducibility. All replicates performed were biological replicates, rather than technical replicates, which represent independent data points. For example, replicate cultures were grown in separate containers and metabolites were measured independently from one another. Microscopy images and chromatograms shown in the paper are representative of 2-3 independent experiments. All attempts at replication were successful.
Randomization	Yeast colonies from transformations were selected randomly. Among the selected ones, further screening was carried out by fermentation to obtain the single one for product fermentation.
Blinding	Blinding was not necessary since measurements did not involve bias from the experimentalist.

Reporting for specific materials, systems and methods

We require information from authors about some types of materials, experimental systems and methods used in many studies. Here, indicate whether each material, system or method listed is relevant to your study. If you are not sure if a list item applies to your research, read the appropriate section before selecting a response.

Materials & experimental systems

n/a	Involved in the study	
☒	☐	Antibodies
☐	☒	Eukaryotic cell lines
☒	☐	Palaeontology
☒	☐	Animals and other organisms
☒	☐	Human research participants
☒	☐	Clinical data

Methods

n/a	Involved in the study	
☒	☐	ChIP-seq
☒	☐	Flow cytometry
☒	☐	MRI-based neuroimaging

Eukaryotic cell lines

Policy information about cell lines

Cell line source(s)	All Yarrowia lipolytica strains used in this study was based on the parental strain po1g that was purchased from Yeastern Biotech.
Authentication	Each Yarrowia lipolytica strain was confirmed by the existence of key genes through colony PCR. Their proper morphology was also confirmed on agar plates and under the microscope.
Mycoplasma contamination	Yarrowia lipolytica strains were not tested for mycoplasma
Commonly misidentified lines (See ICLAC register)	None
